# Amyloid-β specific regulatory T cells attenuate Alzheimer’s disease pathobiology in APP/PS1 mice

**DOI:** 10.1186/s13024-023-00692-7

**Published:** 2023-12-18

**Authors:** Pravin Yeapuri, Jatin Machhi, Yaman Lu, Mai Mohamed Abdelmoaty, Rana Kadry, Milankumar Patel, Shaurav Bhattarai, Eugene Lu, Krista L. Namminga, Katherine E. Olson, Emma G. Foster, R. Lee Mosley, Howard E. Gendelman

**Affiliations:** Department of Pharmacology and Experimental Neuroscience, University of Nebraska Medical Center, Omaha, Nebraska USA

**Keywords:** Treg cell therapy, Alzheimer’s disease, Immunotherapy, Antigen specific, T cell receptor, Amyloid beta

## Abstract

**Background:**

Regulatory T cells (Tregs) maintain immune tolerance. While Treg-mediated neuroprotective activities are now well-accepted, the lack of defined antigen specificity limits their therapeutic potential. This is notable for neurodegenerative diseases where cell access to injured brain regions is required for disease-specific therapeutic targeting and improved outcomes. To address this need, amyloid-beta (Aβ) antigen specificity was conferred to Treg responses by engineering the T cell receptor (TCR) specific for Aβ (TCR_A__β_). The TCR_Ab_ were developed from disease-specific T cell effector (Teff) clones. The ability of Tregs expressing a transgenic TCR_Aβ_ (TCR_Aβ_ -Tregs) to reduce Aβ burden, transform effector to regulatory cells, and reverse disease-associated neurotoxicity proved beneficial in an animal model of Alzheimer’s disease.

**Methods:**

TCR_A__β_ -Tregs were generated by CRISPR-Cas9 knockout of endogenous TCR and consequent incorporation of the transgenic TCR_Ab_ identified from Aβ reactive Teff monoclones. Antigen specificity was confirmed by MHC-Aβ-tetramer staining. Adoptive transfer of TCR_Aβ_-Tregs to mice expressing a chimeric mouse-human amyloid precursor protein and a mutant human presenilin-1 followed measured behavior, immune, and immunohistochemical outcomes.

**Results:**

TCR_Aβ_-Tregs expressed an Aβ-specific TCR. Adoptive transfer of TCR_Aβ_-Tregs led to sustained immune suppression, reduced microglial reaction, and amyloid loads. ^18^F-fluorodeoxyglucose radiolabeled TCR_Aβ_-Treg homed to the brain facilitating antigen specificity. Reduction in amyloid load was associated with improved cognitive functions.

**Conclusions:**

TCR_Aβ_-Tregs reduced amyloid burden, restored brain homeostasis, and improved learning and memory, supporting the increased therapeutic benefit of antigen specific Treg immunotherapy for AD.

**Graphical Abstract:**

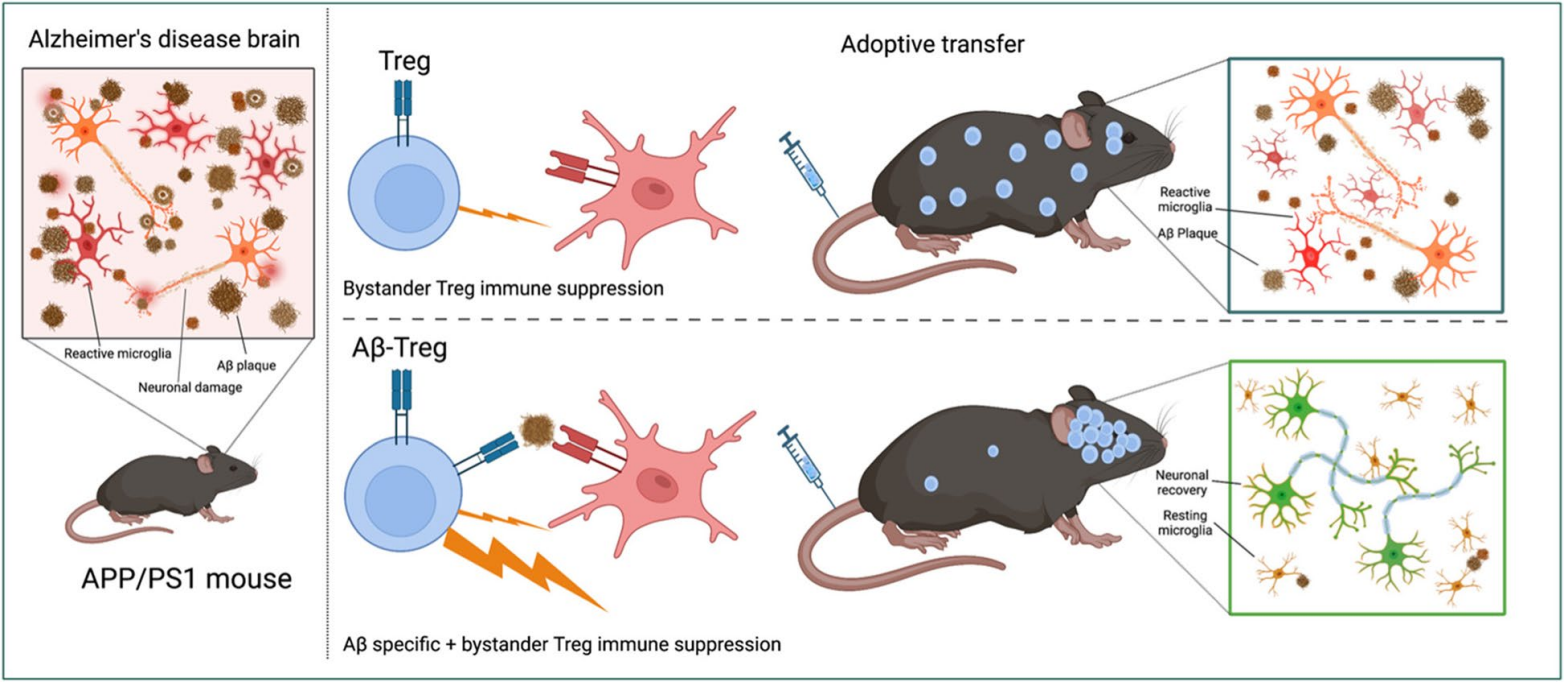

**Supplementary Information:**

The online version contains supplementary material available at 10.1186/s13024-023-00692-7.

## Background

Alzheimer’s disease (AD) is the most common neurodegenerative disorder [1]. Disease is clinically manifest by progressive cognitive decline. Pathologically, disease progression is linked to deposition of extracellular amyloid β (Aβ) plaque deposition, intracellular neurofibrillary tangles, and neuroinflammation [2]. Current drug regimens provide only symptomatic benefit and are ineffective at halting progression of disease [3, 4]. Disease-modifying treatments have used active or passive immunization to clear Aβ plaques. While each proved successful at reducing plaque burden and cognitive defects in animals, [5–7] human trials showed limited success with adverse reactions following immunizations. Study cessation was mandated after active immunization due to the development of meningoencephalitis in a few of the treated patients [8]. This was attributed to the emergence of effector T cells (Teffs) [8–10]. CD4+ Teffs induced by Aβ vaccination produced disease-associated Aβ-specific type-1 T helper (Th1) cells [11].

Thus, the effectiveness of active immunization remains, at present, uncertain. This includes the deployment of Th2-biased adjuvants or by limiting the length of the Aβ epitope to avoid neurotoxic T cell responses [12, 13] [14]. Regrettably, such strategies do not generate stable T cell phenotypes and may elicit undesired Teff responses. The development of optimal neuroprotective immune responses, especially in aged patients, who possess weakened or compromised immune systems remains challenging [15]. Currently, two passive Aβ-specific monoclonal antibody therapies, aducanumab and lecanemab, have US Food and Drug Administration (FDA) approval for AD treatments but have shown more limited success and high prevalence of adverse events due to amyloid-related imaging abnormalities (ARIA) edemas and effusions [16–19].

Given each of these limitations for immunization-based disease-modifying AD therapeutics, T cell and chimeric antigen receptor-based therapies (TCR and CAR-T) are attractive therapeutic alternatives. Notably, T cell-based therapies, using assorted T effector phenotypes, have produced remarkable clinical outcomes in the field of cancer [20, 21]. However, given the adverse events observed with Th1 induction during active immunization strategies, T cell-based therapy for AD required a new directive. This was found through the deployment of regulatory T cells (Tregs). While Tregs are well known to maintain immunological tolerance during disease, such tolerance may be altered. In disease states, Teffs react to misfolded Aβ deposits, clonally expand, then affect neuroinflammation and AD neuropathology [22–25]. Such responses were observed in AD patients before onset of disease symptoms [22, 24, 26, 27]. Our own works have shown that adoptive transfer of Aβ-reactive Teffs accelerate amyloid pathology and cognitive defects in mice expressing chimeric mouse/human amyloid precursor and a mutant human presenilin 1 protein (APP/PS1) [28, 29].

The path forward focused on harnessing CD4+ Tregs for neuroprotection [28]. This novel idea provides a specific immune signature in ameliorating disease due to Treg’s vibrant anti-inflammatory and immunosuppressive activities [28–35]. Treg-inducing agents or, adoptive transfer of polyclonal Tregs confer protection in diverse neurodegenerative diseases. These include but are not limited to AD, stroke, Parkinson’s disease (PD), and amyotrophic lateral sclerosis [28, 35–40]. However, such polyclonal Tregs could also lead to global immune suppression and consequent increased prevalence of infectious or neoplastic diseases. Thus, Tregs specific for disease-inducing pathological proteins such as Aβ, could overcome nonspecific immune dysregulation and be ideal to drive disease-specific Treg therapy which could be expanded further in a wide range of neurodegenerative diseases.

With these goals in mind, we investigated whether Tregs expressing a TCR specific for Aβ (TCR_Aβ_-Tregs) attenuate AD in APP/PS1 mice. We hypothesized that TCR_Aβ_-Tregs target amyloid-rich regions in brain that lead to neuroprotective outcomes. Tregs were specifically engineered for Aβ reactivity using an Aβ-specific TCR identified from Aβ-specific monoclonal Teffs [41]. In an APP/PS1 AD model, we now show that TCR_Aβ_-Tregs target the brain. This results in increased reduction of reactive microglia numbers and composition concomitant with increased amyloid plaque clearance and improved cognitive outcomes.

## Methods

### Knockout of endogenous TCR

Polyclonal Tregs were isolated from non-transgenic mice using the EasySep™ mouse Treg enrichment kit (cat. 18,783, Stemcell Technologies, Cambridge, MA), per the manufacturer’s instructions. Tregs were maintained in culture using 1:1 ratio of mouse T cell activating CD3/CD28 Dynabeads (cat. 11456D, Thermo Fisher Scientific, Waltham, MA) and 1000 IU/mL IL-2 (cat. 212–12, Peprotech, Cranbury, NJ) in complete RPMI-1640 media supplemented with 10% fetal bovine serum (FBS), 2 mM L-glutamine, 25 mM HEPES, 1 mM sodium pyruvate, 1 × nonessential amino acids, 55 nM 2-mercaptoethanol, 100 U/mL penicillin, and 100 μg streptomycin (complete RPMI-1640). The endogenous TCRs of isolated polyclonal Tregs were deleted using CRISPR-Cas9 technology. Guide RNAs (gRNAs) targeting the TCR alpha (U*A*U*GGAUUCCAAGAGCAAUG) and TCR beta (U*G*G*GGUCAGCACGGACCCUC) region and Cas9 nuclease were obtained from Sythego, Redwood city, California. To delete endogenous TCRs, 50 μM (pmoles/μL) of each guide RNA in 1XTris-EDTA (10mMTris, 1mMEDTA, 0.2μ filtered) was mixed with 20 μM (pmoles/μL) Cas9 and electroporated into cultured Tregs using 4D-nucleofector™ X unit (cat. AAF-1003X, Lonza, Basel, Switzerland) according to the manufacturer’s mouse T cell protocol. Briefly, TCR-alpha, and TCR-beta gRNAs (1μL, 100 pmol stock) and Cas9 (2μL, 40 pmol stock) were incubated in a 0.5 mL microcentrifuge tube for 5 min. Simultaneously, 2 × 10^6^ Tregs were collected in a 0.5 mL microcentrifuge tube and resuspended in 16 μL of P3 nucleofector solution (cat. V4XP-3032, Lonza, Basel, Switzerland). The gRNAs preincubated with Cas9 mixture was mixed with Tregs in a total volume of ~ 20 μL. This mixture was transferred to one well in the Primary Cell 4D-Nucleofector™ X Kit S (cat. V4XP-3032. Lonza, Basel, Switzerland) and electroporated using the DN-100 program. Immediately after electroporation, 100 μL warm complete RPMI-1640 media was added to the well and incubated at 37 °C for 15 min to help cells recover. Cells were then collected and plated in 24 well plates with 1:1 Dynabeads and 1000 IU/mL IL-2 in complete RPMI-1640 media. Cells were analyzed for TCR knockout using anti-TCRα/β-PE antibody (cat. LS-C76264, LSBio, Shirley, MA) by flow cytometry. TCR knockout Tregs (TCR^−−^-Tregs) were sorted for anti-TCRα/β-PE negative cells and maintained in 1:1 ratio Dyna beads and 1000 IU/mL IL-2. Sustained deletion of endogenous TCRs was confirmed by flowcytometry for ~ 2–3 weeks.

### Generation of TCR_Aβ_-Treg

To generate TCR_Aβ_-Tregs, a plasmid construct encoding the TCR_Aβ_ previously identified by our laboratory from Aβ-reactive Teff monoclones [41] was electroporated into TCR^**−−**^-Tregs at either 0.5 μL or 0.25 μL of 1.56 μg/μL of the plasmid construct. For the in vitro evaluation of TCR_Aβ_-Tregs, 2 × 10^6^ TCR^−−^-Treg were collected into a 0.5 mL microcentrifuge tubes, resuspended in ~ 20 μL P3 nucleofector solution (cat. V4XP-3032, Lonza, Basel, Switzerland) and mixed with either 0.5 μL or 0.25 μL of 1.56 μg/μL of a TCR_Aβ_ plasmid. The mixture was transferred into the Primary Cell 4D-Nucleofector™ X Kit S (cat. V4XP-3032. Lonza, Basel, Switzerland) and electroporated using the DN-100 program as previously described. For the animal studies, TCR^−−^-Tregs electroporated with 0.5 μL of 1.56 μg/μL of TCR_Aβ_ plasmid construct was used. Incorporation of the TCR_Aβ_ to TCR^−−^-Tregs after electroporation was confirmed using anti-TCRα/β-PE antibody (cat. LS-C76264, LSBio, WA). To confirm Aβ specificity of the engineered Tregs, tetramers of Aβ_1-42_ T cell epitopes presented by H-2^b^ haplotypes were constructed with I-A^b^ and the Aβ amino acid 15–30 (MHCII-IA^b^–KLVFFAEDVGSNKGA) conjugated to fluorophore BV421 (National Institute of Health (NIH) Tetramer Core Facility, Emory University, Atlanta, GA). MHCII-IA^b^–PVSKMRMATPLLMQA tetramer with irrelevant peptide was used as control. For tetramer staining, 3 × 10^5^ TCR_Aβ_-Tregs or polyclonal Tregs were incubated with MHCII-IA^b^–KLVFFAEDVGSNKGA Aβ tetramer (2.4 μg) or MHCII-IA^b^–PVSKMRMATPLLMQA control tetramer (2.4 μg) for 3 h at 37 °C. After incubation, tetramer-stained T cells were reacted with anti-CD3e-PE and anti-CD4-APC-H7 antibodies for 30 min at room temperature, followed by live-dead staining with propidium iodide (0.5 μg/ml) for 5 min at room temperature. Stained T cells were analyzed with a LSR II flow cytometer and FACSDiva Software (BD Bioscience) at the University of Nebraska Medical Center (UNMC) Flow Cytometry Research Facility. To evaluate the cytokine profile of the engineered cells, 1 × 10^6^ TCR_Aβ_-Tregs, polyclonal Tregs, or TCR^−−^-Tregs were stimulated with PMA (20 ng/mL) and ionomycin (1 μM) overnight in complete RPMI-1640 media. Cell supernatants were collected, and cytokine profile evaluated using mouse cytokine array kit (cat. ARY006, R&D systems, Minneapolis, MN) following manufacturer’s instructions. The films were developed and imaged on iBright CL1500 imaging systems (Invitrogen, Waltham, MA) and average signal (pixel density) was analyzed using ImageJ software. For controls, TCR^−−^-Tregs were electroporated with plasmid vector without TCR construct (empty vector, EV) generating EV-Tregs.

### TCR_Aβ_ lentiviral constructs

The lentiviral constructs for transducing the TCR_Aβ_ into target cells were performed as previously described [42]. Lentivirus construct containing the TCR_Aβ_ along with lentiviral packaging mix were co-transfected into HEK293FT cells using Lipofectamine™ 3000 Transfection Reagent (cat. L3000001, Thermofisher, Waltham, MA) according to manufacturer’s instructions. Following incubation, the cell supernatants containing the TCR_Aβ_ lentiviral constructs were purified by passing through 0.45 μm filters and concentrated by ultracentrifugation at 100,000 × g for 1 h. The viral stock titered against HEK293FT cells yielded a titer of 10^9^ transduction units/mL. Transdux-Max (cat. LV860A-1, System Biosciences, Palo Alto, CA) was used for the transduction of the TCR_Aβ_ lentiviral constructs into TCR^−−^-Treg, human PBMC’s, HEK293FT and CEM-SS cells according to manufacturer’s instructions. Successful transduction and expression of TCR_Aβ_ was confirmed by tetramer staining with MHCII-IA^b^–KLVFFAEDVGSNKGA as previously described.

### Adoptive Cell Transfer in APP/PS1 mice

All animal experiments were approved by the Institutional Animal Care and Use Committee of the UNMC. Transgenic mice overexpressing human APP695 with the Swedish mutation (Tg2576) were obtained from Drs. G. Carlson and K. Hsiao-Ashe through the Mayo Medical Venture [43]. PS1 mice overexpressing human PS1 with M146L mutation were provided by Dr. K. Duff from the University of South Florida [44]. Both mice were maintained on the B6;129 hybrid background. Male Tg2576 mice were crossbred with female PS1 mice to generate APP/PS1 double-transgenic mice and non-transgenic (non-Tg), and B6;129 mice were developed in parallel, as described previously [35, 45–47]. Female APP/PS1 mice, 8 months old, and age-matched non-transgenic littermates were blindly randomized into different experimental groups. Either 1 × 10^6^ TCR_Aβ_-Tregs, polyclonal Tregs, or EV-Tregs (TCR^−−^-Tregs electroporated with empty plasmid vector) in 100 µL phosphate-buffered saline (PBS) were adoptively transferred to APP/PS1 recipient mice, intravenously via tail vein using a 28-gauge needle affixed to a sterile tuberculin syringe, thrice at 1-week intervals. Both the engineered Tregs (TCR_Aβ_-Tregs and EV-Tregs) and polyclonal Tregs were maintained and amplified using 1:1 ratio of anti-CD3/CD28 Dynabeads and 1000 IU/mL IL-2 prior to adoptive transfer. Both age-matched untreated APP/PS1 mice and non-transgenic mice served as controls.

### Radial arm water and Y-maze tests

After the third adoptive cell transfer, mice were submitted for radial arm water maze (RAWM) testing in a blinded fashion to assess memory impairment as previously described [35, 48]. Briefly, mice from masked cages were introduced into the circular water filled tank (diameter-110 cm and height-91 cm, San Diego Instruments) with triangular inserts that produce six swim paths radiating from the center. Special cues are fixed on the tank wall to guide mouse orientation. At the end of any one arm, a circular plexiglass hidden platform (diameter-10 cm) is submerged 1 cm beneath the water level. The platform was placed in the same arm for four consecutive acquisition trials (T1–T4), and retention trial (T5), but in a different arm on different experimental days. For T1–T4, the mouse started the task from a randomly chosen arm without a platform. After four trials, the mouse was returned to its cage for 30 min and reintroduced into the T4 arm, for the delayed retention trial (T5). Each trial lasted 1 min, and an error was scored when the mouse entered the wrong arm; entered the arm with the platform, but did not climb on it; or did not make a choice for 20 s. The trial ended when the mouse climbed and stayed on the platform for at least 10 s. The mouse was allowed to rest on the platform for 20 s between trials. If the mouse did not climb the platform, after 60 s, it was gently guided to the submerged platform. The T1, T4 and T5 trial errors over 9-day test were divided into three blocks (block-1 days 1–3, block-2 days 4–6, block-3 days 7–9), and the errors in each block were averaged for statistical analysis. We used the Y-maze test to evaluate spatial learning and memory using the short-term alterations method [49]. The arms of the maze had 39.5*8.5*13 cm dimensions with 120° angle between the arms. Mice were allowed to explore the maze freely for 8 min, and the total entries were recorded visually. Successful entries were defined as consecutive entries into three different arms.

### ^18^F-FDG cell tracking

To confirm migration and accumulation of Tregs to the brain, TCR_Aβ_-Tregs or polyclonal Tregs were radiolabeled with ^18^F-fluorodeoxyglucose (^18^F-FDG, Cardinal Health, Omaha, NE). Briefly, cells were glucose starved by incubating them in glucose-free RPMI-1640 media (cat. 11,879–020, Gibco-Thermo Fisher Scientific, Waltham, MA) for 4 h at 37 °C. Following starvation, cells were incubated with a previously optimized non-toxic concentration, 1 mCi/mL ^18^F-FDG, for 1 h at 37 °C to allow uptake of radioactive glucose. Neither the 4 h glucose starvation nor the 1 mCi/mL ^18^F-FDG had a marked effect on cell viability. Cells were then washed thrice to remove excess radioactive compound and 5 × 10^6^ cells/mouse were injected intravenously via the tail vein to 8-month-old APP/PS1 and allowed 10 min for uptake. Radioactivity was measured using combined positron emission tomography (PET/CT) (β-Cube, Molecubes Inc., Lexington, MA) at 0.5, 2, 4 and 6 h in the UNMC PET Core Facility. Briefly, mice were anesthetized with 2% isoflurane with oxygen. Acquisition time of 10 min was used at time points 0.5, 2, 4, and 6-h post-injection to measure radioactivity. Computed tomography (CT) scans were acquired using TriFoil imaging Triumph (Tri-foil imaging Northridge, CA). The X-ray tube was used at 150 μA and 75 kV. Each run obtained 512 projections with an exposure time of 230 ms. VIVOQUANT software (inviCRO, Boston, MA) was used to overlay and analyze CT and PET reconstructed images. The 3D brain atlas software was used for quantifying radioactivity from different brain regions.

### Brain glucose uptake

Mice were fasted overnight and ^18^FDG (Cardinal Health, Omaha, NE) was injected intravenously to fasted mice, and brain glucose uptake was evaluated by PET scan. Briefly, mice were anesthetized by 2% isoflurane along with oxygen. ^18^FDG with an activity of 70 µCI in a total volume of 0.1 ml PBS was intravenously injected into the lateral tail vein and allowed for 10 min of uptake. At 30 min post-injection, 10 min PET acquisitions were carried out using Molecube beta-CUBE (MOLECUBES NV, Gent, Belgium). CT scans were acquired using TriFoil imaging Triumph (Tri-foil imaging Northridge, CA). The X-ray tube was used at 150 μA and 75 kV. Each run obtained 512 projections with an exposure time of 230 ms. VIVOQUANT software (inviCRO, Boston, MA) was used to overlay the CT and PET reconstructed images for glucose uptake measurements.

### Measures of Treg function

Comparison of Treg function of the engineered Treg cells or systemic Tregs from different mice treatments was performed as described earlier [50]. For evaluating systemic Treg function, Tregs (CD4+ CD25+) and Tresp (T responder) (CD4+ CD25−) cells were isolated from the mice spleens using EasySep™ mouse Treg enrichment kit (Cat. 18,783, Stemcell Technologies, Vancouver, CA), per the manufacturer’s instructions, and CD4+ T cells were enriched from splenic single cell suspension by negative selection using the EasySep™ mouse CD4+ T cell isolation cocktail. From the enriched CD4+ populations, CD25+ cells were positively selected using the EasySep™ mouse CD25 + Treg selection cocktail. The isolated CD4+ CD25+ cells were more than 97% FOXP3+ as determined by flow cytometric analysis. The CD4+ CD25− Tresps, more than 96% pure, were collected from naïve non-Tg mice spleens and used in the proliferation assay. Briefly, Tresp cells were labeling with carboxyfluorescein succinimidyl ester (CFSE) (Cat. C34554, Thermo Fisher Scientific, Waltham, MA). CD4+ CD25+ Tregs from different treatment groups were serially diluted in a U-bottom 96-well plate to obtain 50, 25, 12.5, and 6.25 × 10^3^ Tregs in 100 µl of media followed by addition of 50 × 10^3^ CFSE-labeled Tresp cells from non-Tg mice into each well to obtain Treg:Tresp ratios of 1:1, 0.5:1, 0.25:1 and 0.125:1. Wells with only Tresps served as controls. Mouse T cell activating CD3/CD28 Dynabeads (Catalog no. 11456D, Thermo Fisher Scientific) were added to each well at a bead:Tresp ratio of 1:1 to induce Tresp proliferation. Tresp cells alone with and without Dynabeads served as controls for baseline Tresp proliferation without Treg suppression. The immunosuppressive function of Tregs to inhibit proliferation of CFSE-stained Tresps was determined after 72 h incubation at 37 °C using flow cytometric analysis and is reported as Treg-mediated % inhibition: [1-(Percent proliferation of Tresp:Treg dilution ÷ Percent proliferation of stimulated Tresp alone)] × 100. For comparing Treg function of the engineered Tregs, ex-vivo cultured Tregs or TCR_Aβ_-Treg collected on day 2 after electroporation of TCR^−−^-Tregs with 0.25 μL or 0.5 μL of 1.56 μg/mL the TCR_Aβ_ plasmid were co-incubated with CFSE-labeled Tresp cells from non-Tg mice at a ratio of 1:1, 0.5:1, 0.25:1 and 0.125:1 Treg:Tresp for 72 h days and suppressive function was evaluated by flowcytometry as described earlier.

### Antigen (Aβ) specific treg function

To evaluate antigen mediated Treg function of the engineered TCR_Aβ_-Treg cells, CFSE labelled Tresp cells isolated from non-Tg mice were stimulated overnight with CD3/CD28 Dynabeads at 1:1 bead:Tresp cell ratio in a 48 well plate. Dynabeads were magnetically removed to isolate pre-stimulated CFSE-labeled Tresp cells. In a 96 well U bottom plate, 50 × 10^3^ pre-stimulated CFSE-labeled Tresp were co-incubated with an equal number of ex-vivo cultured Tregs, or TCR_Aβ_-Tregs collected on Day-2 post electroporation with 0.5 μL of 1.56 μg/mL TCR_Aβ_ plasmid. For antigen stimulation, 5μL of Aβ-MHC tetramer (1.16 mg/mL) was added per well and incubated for 72 h. Pre-stimulated CFSE labelled Tresp alone or un-stimulated CFSE-labeled Tresp were used as controls for baseline Tresp proliferation. The antigen mediated immunosuppressive function of Tregs to inhibit proliferation of pre-stimulated CFSE-stained Tresp cells was determined using flow cytometric analysis and is reported as Treg-mediated percent inhibition: [1-(percent proliferation of 1:1 Tresp:Treg dilution ÷ percent proliferation of pre-stimulated Tresp alone)] × 100.

### Flow cytometry

On day of sacrifice, pentobarbital was used to terminally anesthetize the mice. Spleens were harvested into complete RPMI-1640 media and blood collected by cardiac puncture in K_3_EDTA tubes (cat. 450,475, Greiner BioOne North America, Monroe, NC). Mice were then pericardially perfused with PBS, and brains and lymph nodes (axial, cervical and inguinal) were harvested. To isolate immune cells from brain, brain tissues were homogenized in Hanks’ balanced salt solution (HBSS) and passed through a 70 μm cell strainer. Cells were centrifuged and resuspended in 500μL HBSS and incubated with 100 μL DNAase and 100 μL collagenase for 5 min at 37 °C. After incubation, cells were centrifuged, resuspended in 4 mL 30% Percoll, layered with 4 mL HBSS, centrifuged (700xg for 10 min), and the cell pellet collected for flow staining. Single cell suspensions of splenocytes (10^6^ cells), lymph nodes (10^6^ cells), or whole blood (50 μL) were used for flow staining. To determine the frequency of Tregs in different organs, 10^6^ cells were incubated at room temperature for 30 min in 100 μL PBS/1% BSA/0.09% NaN3 with PE-anti-CD3e (cat. 12–0031-81, Invitrogen, Waltham, MA), APC-H7-anti-CD4 (cat. 560,181, BD Pharmingen), PE-Cyanine5.5-anti-CD8a (cat.35–0081-82, Invitrogen), PE-Cy7-anti-CD25 (cat. 25–0251-82, eBioscience, San Diego, CA), and Alexa Fluor 488-anti-FOXP3 (cat. 320,012, BioLegend, San Diego, CA). Isotypes and FMO (fluorescence-minus-one) were used for accurate gating. To determine the frequency of Aβ reactive CD4+ T cells, 1 × 10^6^ lymph node cells were stimulated with Aβ_1–42_ (25 μg/ml) in presence of feeder cells (irradiated splenocytes) and IL-2 (20 IU/mL) for 5 days at 37 °C. On day 5, cells were collected by centrifugation and incubated Live/Dead™ Fixable Blue Dye (cat. L23105, Thermo Fisher Scientific) followed by MHCII-IA^b^–KLVFFAEDVGSNKGA Aβ tetramer (6 µg) or MHCII-IA^b^–PVSKMRMATPLLMQA control tetramer (6 µg) for 3 h at 37 °C. After incubation, live/dead stained T cell-MHCII-Aβ tetramer complexes were stained with PE-anti-CD3e, APC-H7-anti-CD4, PE-Cyanine5.5-anti-CD8a, PE-, and Alexa Fluor 488 labeled anti-FOXP3 for flow cytometric analysis.

### Immunohistochemistry

After transcardial perfusion, brains were immediately harvested and divided into two hemispheres. The left was immediately frozen on dry ice for biochemical analysis and the right was immersed in fresh, depolymerized 4% paraformaldehyde in PBS for 48 h at 4 °C and cryoprotected by immersion in 15% then 30% sucrose for 24 h/immersion at 4 °C. Fixed brains were sectioned coronally with a cryostat (Thermo Fisher Scientific), and 30 μm sections were serially collected and stored at − 80 °C. Immunohistochemistry was performed using antibodies against pan-Aβ (1:500, rabbit polyclonal, cat. 715,800, Thermo Fisher Scientific), Iba1 (1:1000, rabbit polyclonal, cat. 01919741, Wako Chemicals, Richmond, VA) and doublecortin (Dcx) (1:500, goat polyclonal, cat. Sc8066, Santa Cruz Biotechnology, Dallas, TX). For immunodetection, biotin-conjugated anti-rabbit or anti-goat IgG secondary antibody was used followed by a tertiary incubation with Vectastain ABC Elite kit (cat. PK6100, Vector Laboratories, Newark, CA). One percent thioflavin-S in 50% ethanol was used for counterstaining of compact amyloid plaque (cat. T1892, Sigma-Aldrich, St. Louis, MO). For each of the immunohistochemical staining, six sections/slide were collected at eight intervals and were used for each of the experimental groups. Slides were masked and coded, and Aβ occupied area was calculated using Cavalieri estimator probe (grid spacing 15 μm), while the number of Iba1-reactive microglia cells were counted using the Optical Fractionator probe of Stereo Investigator system (MBF Bioscience, Williston, VT) as described earlier [45]. Briefly, a high-sensitivity digital camera (OrcaFlash2.8, Hamamatsu C11440-10C, Hamamatsu, Japan) interfaced with a Nikon Eclipse 90i microscope (Nikon, Melville, NY, USA) was used. Within the Stereo Investigator, the contour in each section was delineated using a tracing function. While sections showed tissue shrinkage along the anteroposterior axis, the extent of shrinkage between sections from different animals was similar. The dimensions for the counting frame (120 × 100 μm) and the grid size (245 × 240 μm) were set. The z-plane focus was adjusted at each section for clarity. Immunoreactive cells were marked positive in each counting frame and quantified by the software based on the section parameters and marked cell counts.

### Aβ detection by ELISA

Snap-frozen mouse cortex was homogenized in 50 mM Tris–HCL (pH 7.6) containing 150 mM of NaCl and a protease inhibitor. Lysates were centrifuged at 20,000 × g for 60 min at 4 °C, and the supernatants were collected for detecting soluble fraction of Aβ_42_. For detecting insoluble fractions of Aβ_42_, pellets were dissolved using 6 M guanidine-HCL and were centrifuged at the same speed and time at room temperature. Aβ_1-42_ loads in the brain cortex were quantified using an ELISA kit (Quantikine ELISA, cat. DAB142, R & D Systems, Minneapolis, MN) following the manufacturer's protocol.

### RNA extraction and qPCR

RNA was extracted from the brain cortexes using RNeasy Mini Kit (Qiagen, cat 74,101, Hilden, Germany) following the manufacturer’s protocol. Extracted RNA was quantified using NanoDrop One (Thermo Scientific, cat ND-ONE-W, Waltham, MA). For cDNA preparation, 1ug RNA was reverse transcribed using a TaqMan reverse transcription kit (Applied Biosystems, cat N808080234, Waltham, MA). Prepared cDNA was diluted 1:5 for downstream qPCR assay. For the qPCR assay, TaqMan Gene Expression Master Mix kit (Applied Biosystems, cat 4,369,016, Waltham, MA) was used to quantify the relative expression of ITGAX, Clec7A, GFAP, and TREM2 genes. Similarly, RPLP0 gene was used as the reference gene. Commercially available predesigned murine primers were purchased from Integrated DNA Technologies, Coralville, IA (Primer sequences provided in Supplementary data, Table 2). Relative gene expression was calculated using the delta-delta cycle threshold method (2 − ΔΔCt) [51].

### Statistical analysis

All data were normally distributed and presented as mean values ± standard errors of the mean (SEM). Comparisons of means between groups were analyzed by one-way ANOVA or two-way repeated measures ANOVA followed by Turkey’s post hoc test using GraphPad Prizm software version 8.0 (GraphPad Software, San Diego, CA). A value of *p* ≤ 0.05 was regarded as a significant difference.

## Results

### Generation of Aβ-specific Tregs

**Fig. 1:**
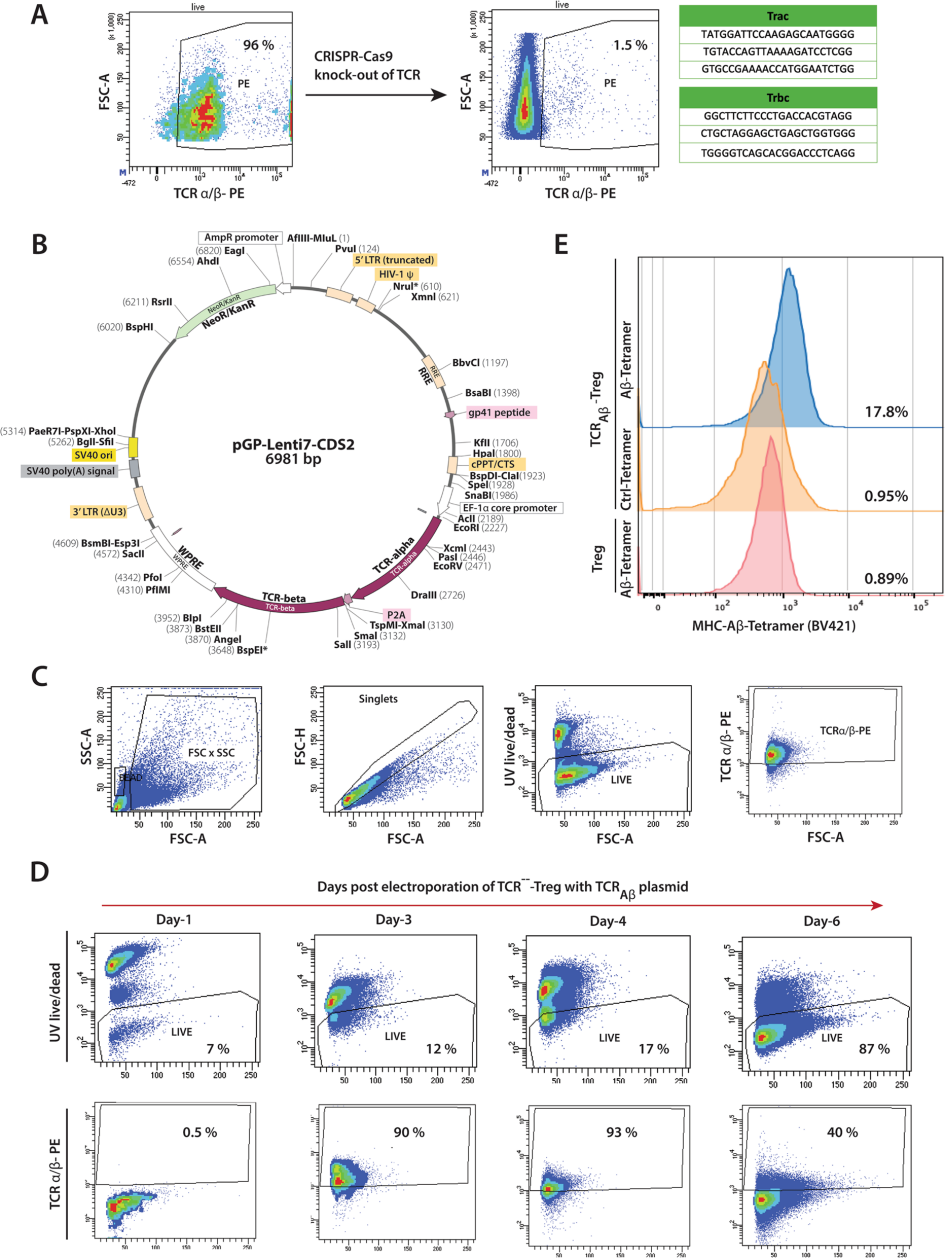
*Generation of TCR*_*Aβ*_*-Tregs*. **A**. Flow cytometry gating confirming the CRISPR-Cas9-mediated knockout of endogenous T cell receptors (TCRs) to generate TCR^−−^-Tregs. Nucleotide sequences represent the guide RNAs targeting the alpha and beta regions of the TCR. **B**. Plasmid design for TCR_Aβ_ electroporation. **C**. Representative gating strategy confirming the TCR expression by TCR-Tregs electroporated with 0.5 μL of TCR_Aβ_ plasmid (plasmid conc. = 1.5 μg/μL). **D**. Time course of TCR_Aβ_ expression on engineered TCR_Aβ_-Tregs post electroporation with TCR_Aβ_ plasmid. Phenotype characterization of engineered TCR_Aβ_-Tregs shown in Supplementary Fig. 3. E. MHCII-IA.^b^-KLVFFAEDVGSNKGA (Aβ T cell epitope) tetramer binding confirming the Aβ reactivity of engineered TCR_Aβ_-Tregs incubated with Aβ-tetramer (blue) compared to TCR_Aβ_-Tregs incubated with control-tetramer (orange) or polyclonal Tregs incubated with Aβ-Tetramer (red)

Our prior studies demonstrated the pathobiological role of Aβ-specific T effector cells (Aβ-Teffs) in APP/PS1 mice. The high-affinity Aβ-Teff clones were generated following immunization of mice with Aβ_1–42_ [41]. The TCR identified from Aβ-Teff clones were used to design TCR_Aβ_ plasmid constructs for lentiviral transduction of Treg recipient cells. As a first step toward engineering, the endogenous TCRs of polyclonal Treg primary isolates were deleted by CRISPR-Cas9 technology. Guide RNAs (gRNAs) encoding the α- and β- chains of the TCR were electroporated into polyclonal Tregs isolated from non-transgenic mice (Fig. 1A). This resulted in the deletion of TCRs on more than 95% of Tregs. TCR knockout Treg cells (TCR^−−^-Tregs) were flow-sorted and the stability of TCR deletions was confirmed by flow cytometric analysis every week for over a month. As a first step for transduction, the lentiviral approach was used to facilitate TCR_Aβ_ entry into TCR^−−^-Tregs. The produced lentiviral construct was able to transfect human PBMC’s, CEMSS and HEK-293 cells with the TCR_Aβ_ (Fig S1,2). However, as lentiviruses poorly transduce mouse T cells, stable transduction of mouse Tregs with TCR_Aβ_ lentiviral constructs was not successful (Fig S1). To overcome this limitation, TCR_Aβ_ encoding plasmids (Fig. 1B) were electroporated into TCR^−−^-Tregs to generate TCR_Aβ_-Tregs. While electroporation of the TCR_Aβ_ encoding plasmid led to significant cell death within 24 h, surviving cells recovered by day-2 and flow cytometric analysis showed stable expression of the TCR for 4–6 days following electroporation (Fig. 1C, D). Aβ specificity of the TCR_Aβ_ was confirmed by flow cytometry of the TCR_Aβ_-Tregs and increased staining of MHCII-IA^b^–KLVFFAEDVG-SNKGA tetramer (Fig. 1E).

**Fig. 2:**
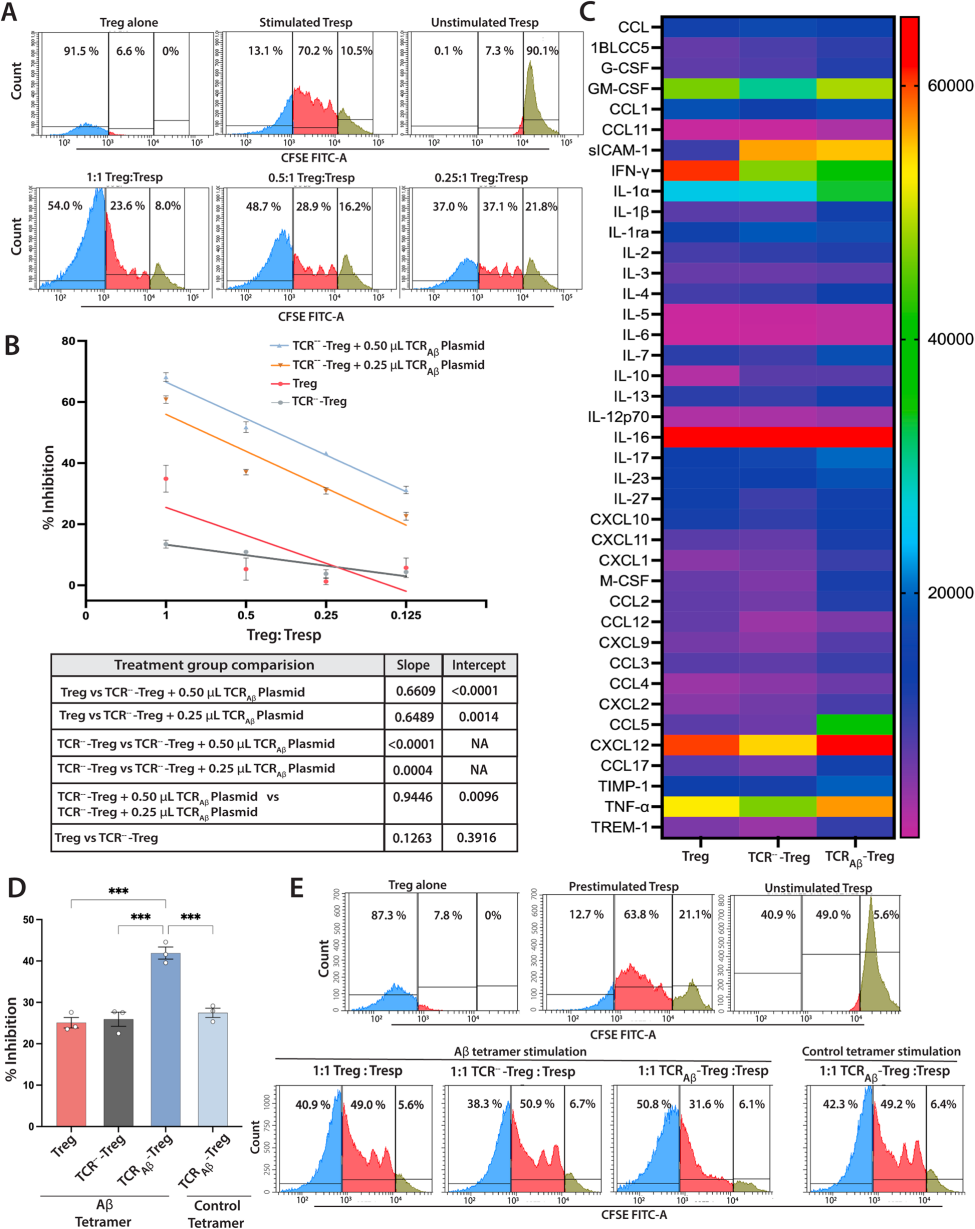
*Characterization of TCR*_*Aβ*_*-Treg immunosuppressive function.* **A**. Representative histograms of Treg suppressive function assay. CFSE-labelled-Tresp (CD4+ CD25-) cells co-incubated (3 days) with decreasing ratio of Treg:Tresp cells in the presence of anti-CD3/CD28 Dynabeads. Undivided CFSE+ Tresp (green peaks), divided CFSE+ Tresp (red peaks), Treg cells (blue peaks). **B**. Quantitation of the immunosuppressive function of TCR_Aβ_-Tregs generated by electroporation of TCR^−−^-Tregs (TCR knockout Tregs) with 0.25 μL or 0.5 μL of TCR_Aβ_ plasmid (plasmid conc. = 1.5 μg/μL). Engineered TCR_Aβ_-Tregs, Tregs or TCR knockout Treg (TCR^−−^-Tregs) were co-cultured with CFSE + Tresp cells (50 K cells/well) from non-Tg mice in the presence of anti-CD3/CD28 Dynabeads. Treg mediated immune suppression (%Inhibition) = [1- (% proliferation of Tresp:Treg dilution ÷ % proliferation of stimulated Tresp alone)] × 100. Linear regression analysis indicates r^2^ > 0.90, *p* < 0.03 for TCR^−−^-Tregs electroporated with 0.25 μL or 0.5 μL of TCR_Aβ_ plasmid. Regression of polyclonal Tregs were r^2^ > 0.50, *p* < 0.03. Table contains *p*-values for slopes and intercepts of Treg functions compared by linear regression analysis, *n* = 3. Data presented as mean ± SEM **C**. Supernatants of Treg (polyclonal), TCR^−−^-Tregs (TCR knockout Tregs), and TCR_Aβ_-Tregs (TCR^−−^-Tregs + 0.5 μL TCR_Aβ_ plasmid) stimulated with PMA/ionomycin assessed by mouse cytokine array. Data represents mean intensities and statistical differences determined by two-way ANOVA tabulated in Supplementary Table 1. D. Quantification of Aβ-mediated Treg suppressive function. CFSE + Tresp cells from non-Tg mice were stimulated overnight with anti-CD3/CD28 Dynabeads. Pre-stimulated CFSE + Tresps were co-cultured with TCR _Aβ_-Tregs (TCR^−−^-Tregs + 0.5 μL TCR _Aβ_) or Tregs (polyclonal) or TCR knockout Treg (TCR^−−^-Tregs) at 1:1 ratio (50,000 cells/well) for 3 days with only MHC-Aβ-tetramer or control-tetramer. Treg–mediated % inhibition was calculated, and statistical differences were determined by one-way ANOVA followed by Turkey’s post hoc test. ***p < 0.001, *n* = 3. Data presented as mean ± SEM **E.** Representative histograms of Aβ-mediated Treg suppressive function of engineered Tregs co-incubated with pre-stimulated CFSE+ Tresp cells in the presence of Aβ-tetramer or control-tetramer. Undivided CFSE+ Tresp (green peaks), divided CFSE + Tresp (red peaks), Treg cells (blue peaks)

To assess the functionality of TCR_Aβ_-Tregs, we determined their ability to suppress proliferation of T responder (Tresp) cells. The Tresp suppressive function of Aβ-Treg was evaluated by co-incubating them with CSFE-labeled Tresp cells in the presence of Dynabeads (mouse T-cell activator CD3/CD28). Knocking out the TCR (TCR^−−^-Tregs) showed a non-significant reduction in ability to suppress Tresp cell proliferation compared to polyclonal Treg (Fig. 2B). TCR_Aβ_-Tregs showed a significant increase in the ability to suppress Tresp cell proliferation compared to polyclonal Tregs (Fig. 2A,B). The increase in suppressive function was dose-dependent on the amount of TCR_Aβ_ plasmid electroporated. The TCR_Aβ_-Tregs that received 0.50 μL of TCR_Aβ_ plasmid showed higher suppressive function compared to those that received 0.25 μL of the plasmid. To further confirm the Aβ-specific Treg suppressive function, a modified Treg function assay was performed where CFSE-labeled Tresps were pre-stimulated with Dynabeads overnight, the beads removed, and the stimulated Tresps co-cultured with TCR_Aβ_-Tregs for three days in the presence of Aβ-tetramer alone as stimulant. Polyclonal Tregs showed similar suppression of Teff cells as compared to TCR^−−^-Treg (Fig. 2D,E). TCR_Aβ_-Tregs showed a significant increase in Aβ-tetramer-dependent Teff suppressive function compared to polyclonal Tregs (Fig. 2D,E). Further, we looked at the cytokine profile of the engineered TCR_Aβ_-Tregs stimulated with PMA and ionomycin using a mouse cytokine array kit. Compared to polyclonal Tregs, TCR_Aβ_-Tregs produced increased Th2-polarizing cytokines (Fig. 2C, Supplementary Table 1). Further, TCR_Aβ_-Tregs showed increased secretion of IL-4 which supports diminished production of IFN-γ. Further chemokines CCL2 and CCL5 that are involved in Treg recruitment in vivo were also elevated (Fig. 2C, Supplementary Table 1).

### Adoptive transfer of TCR_Aβ_-Tregs improves memory formation.

**Fig. 3:**
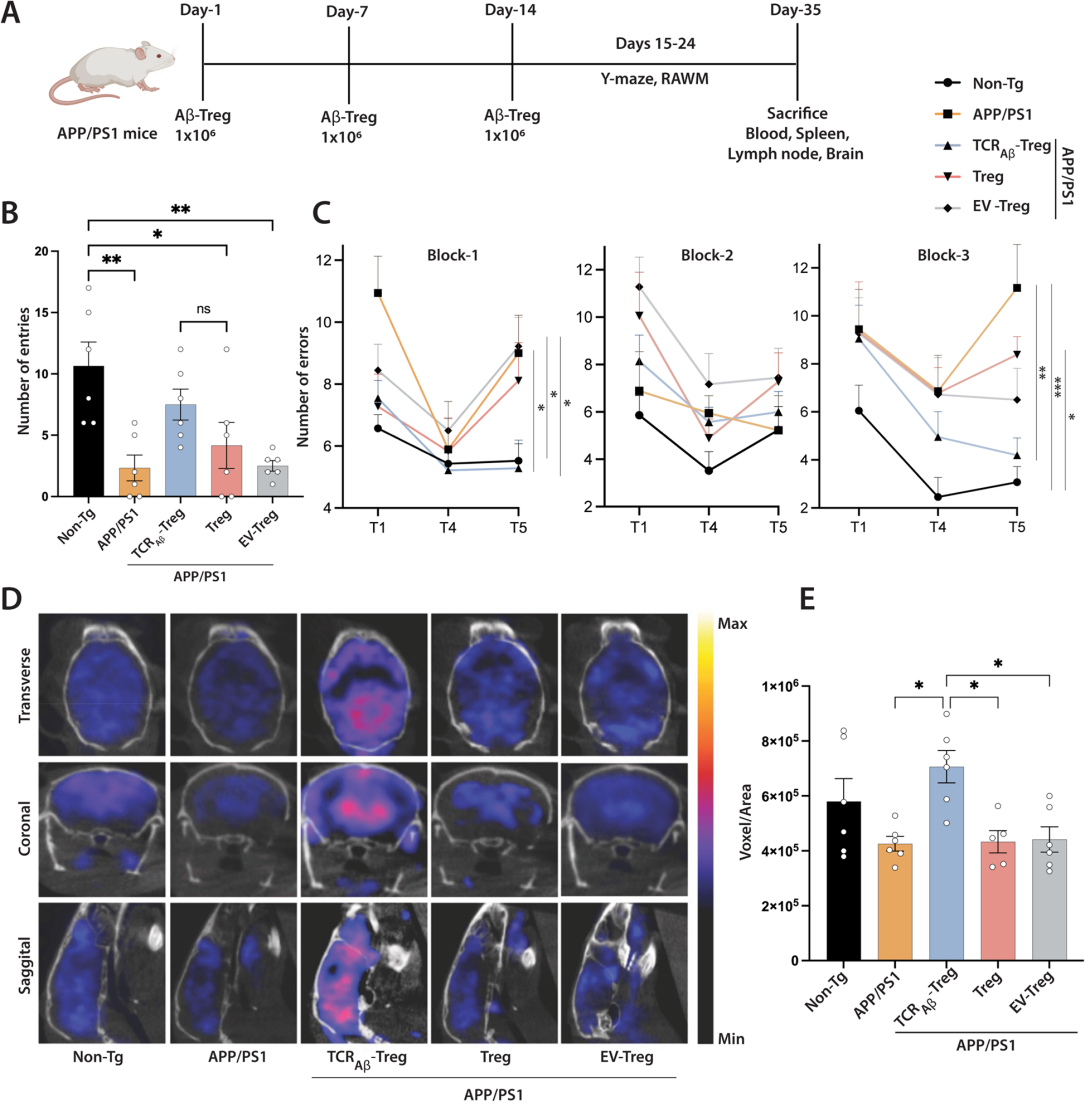
*Adoptive transfer of TCR*_*Aβ*_*-Tregs improve memory function in APP/PS1 mice*. A. Experimental timeline of adoptive transfer experiments. Eight-month-old non-Tg mice (*n* = 6) were untreated, and age-matched APP/PS1 mice were untreated or treated with 1 × 10^6^ TCR_Aβ_-Tregs, polyclonal Tregs (Treg), or EV-Tregs (TCR^−−^-Tregs electroporated with empty plasmid vector). **B** Y maze test performed on experimental mice after adoptive transfers to evaluate spontaneous alteration in mice freely exploring each arm of the Y maze over eight minutes. Successful entries were defined as consecutive entries into three different arms and the number of entries/mice were recorded (*n* = 6). **C.** Radial arm water maze (RAWM) test performed with experimental mice after adoptive transfers. After four trials (T1-T4) the mice were returned their cages for 30 min and reintroduced into the T4 arm for the delayed retention trial (T5). Each trial lasted for 1 min and errors were scored when the mice entered the wrong arm or entered the arm without climbing the platform or didn’t make a choice for 20 s. The trial ended when the mice climbed and stayed on platform for at least 10 s. Errors of 9-day trial were divided into three blocks: Block-1 (days 1–3), Block-2 (days 4–6), Block-3 (days 7–9). The errors in each block were averaged for statistical analysis (*n* = 6). **D** Representative ^18^F-FDG PET images of brain glucose uptake in different treatment groups on the day of sacrifice. **E** Quantification of ^18^F-FDG PET brain glucose uptake on the day of sacrifice (*n* = 5—6). **B**, **C**, and **E** Data presented as mean ± SEM. One-way ANOVA followed by Turkey’s post hoc test was used to determine significant differences between experimental groups. *p < 0.05, **p < 0.01, ***p < *0.001*

We previously showed that adoptive transfer of 1 × 10^6^ monoclonal Aβ-specific Teff clones accelerates memory impairment [41]. As the TCR_Aβ_-Tregs generated via electroporation only transiently express TCR_Aβ_, but are functionally capable, we adoptively transferred 1 × 10^6^ cells once a week, for 3 weeks and evaluated the mice for spatial learning and memory in both the radial arm water maze (RAWM) and Y maze test. These tests were performed a day after the final adoptive cell transfer. In the Y maze test, the APP/PS1 mice demonstrated memory impairment with a significant (*p* < 0.01) reduction in number of arm entries compared to non-transgenic mice. Only APP/PS1 mice treated with TCR_Aβ_-Tregs showed increased number of arm entries. While number of entries did not reach significance compared to untreated APP/PS1 mice, they were not statistically different from those of non-Tg mice compared against Treg or EV-Treg treated APP/PS1 mice (Fig. 3A, B). APP/PS1 mice treated with polyclonal Tregs or EV-Tregs (TCR^--^-Treg electroporated with empty plasmid vector) were not different from untreated APP/PS1 mice in tested memory outcomes.

In the RAWM test, the APP/PS1 mice showed signs of memory impairment as evidenced by significant increases in the numbers of errors in late retention trial T5 (*p* < 0.05 in block-1 and *p* < 0.001 in block-3) compared to non-transgenic mice (Fig. 3C). Notably, treatment of APP/PS1 with TCR_Aβ_-Tregs showed significant improvement in memory outcomes as demonstrated by significantly reduced errors in late retention trial T5 (*p* < 0.05 in block 1 and *p* < 0.01 in block 3) compared to untreated APP/PS1 mice. APP/PS1 mice treated with either polyclonal Tregs or control EV-Tregs did not show any improvement in memory outcomes in the RAWM test. Overall, the results demonstrate that treatment with amyloid β-specific TCR_Aβ_-Tregs improves memory outcomes in APP/PS1 mice compared to treatment with polyclonal Tregs.

Brain glucose hypometabolism is a prominent feature of AD. Improvement in brain glucose uptake and metabolism is a biomarker for memory improvement [49, 50]. ^18^F-FDG PET imaging is commonly used for the diagnosis of dementia states in AD patients and in preclinical animal models [52, 53]. Compared to non-transgenic mice, APP/PS1 mice showed reduced brain glucose uptake (Fig. 3D and E). Treatment of APP/PS1 mice with TCR_Aβ_-Tregs showed significant increase (*p* < 0.05) in glucose uptake compared to APP/PS1 mice that were untreated or treated with polyclonal Tregs or EV-Tregs. Treatment with either polyclonal Tregs or EV-Tregs did not show significant improvement in brain glucose uptake compared to untreated APP/PS1 mice. Together, increased brain glucose uptake after treatment with TCR_Aβ_-Tregs parallels the improved memory outcomes in the Y maze and RAWM tests.

### TCR_A__β_-Tregs facilitate Treg homing to the brain.

**Fig. 4:**
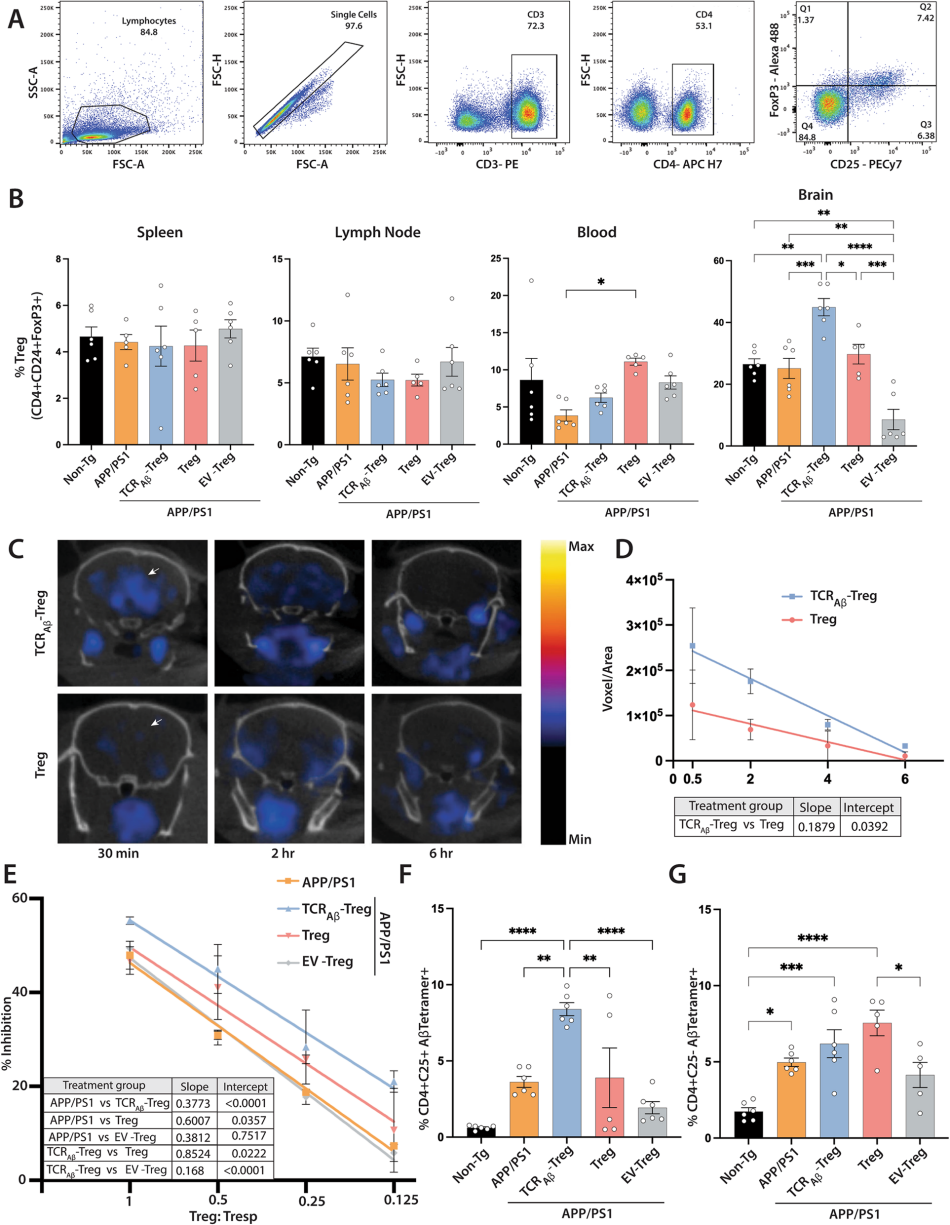
*Adoptive transfer of TCR*_*Aβ*_*-Tregs increase Treg homing to brain and Treg function*. **A** Representative flow cytometric gating strategy for quantification of total Treg frequencies (CD4+ CD25+ FoxP3+). **B** Frequencies of Tregs in spleen, lymph node, blood, and brain in APP/PS1 mice adoptively transferred with three, weekly doses of 1 × 10^6^ TCR_Aβ_-Tregs, polyclonal Tregs (Treg) or EV-Tregs (TCR^−−^-Tregs electroporated with empty plasmid vector) (*n* = 5-6) **C** Representative PET images of APP/PS1 brains after adoptive transfer of ^18^F-FDG radiolabeled polyclonal Tregs (Treg) or TCR_Aβ_-Tregs and their biodistribution evaluated at 0.5, 2, and 6 h. **D** Quantitation of radioactivity from PET images of ^18^F-FDG radiolabeled Tregs or TCR_Aβ_-Tregs in the brain acquired at 0.5, 2, 4, and 6 h (*n* = 3). **E** Suppressive capability of peripheral Tregs from APP/PS1 mice that were untreated or treated with TCR_Aβ_-Tregs, polyclonal Tregs (Treg) or EV-Tregs (TCR^−−^ -Tregs electroporated with empty plasmid vector). Linear regression analysis indicates r.^2^ > 0.90, *p* < 0.001 for all treatment groups. Table contains p-values for slopes and intercepts of compared linear regression analysis (*n* = 3). Flow cytometric analysis of; **F** MHC-Aβ-tetramer positive Tregs (CD4+ CD25+ Aβ-Tetramer+) and **G** MHC-Aβ-tetramer positive CD4+ CD25-Aβ-Tetramer + splenocytes stimulated with Aβ protein and low dose IL-2, (*n* = 5–6). **B**,** F** Data presented as mean ± SEM. One-way ANOVA followed by Turkey’s post hoc test was used to determine significant differences between experimental groups. *p < 0.05, **p < 0.01, ***p < 0.001, ****p < *0.0001*

Our central hypothesis is that given the Aβ reactivity of engineered Tregs, those Tregs will migrate to and accumulate in amyloid rich brain regions. These cells would then generate neuroprotective anti-inflammatory outcomes. Initially to evaluate this, we assessed the distribution of total Tregs (CD4+ CD25+ FoxP3+) by flow cytometry (Fig. 4A) in the spleen, lymph nodes, blood, and brain of WT mice, untreated APP/PS1 mice, or APP/PS1 mice recipients treated with TCR_Aβ_-, polyclonal-, or EV-Treg. Mice were sacrificed 2 weeks after the final adoptive transfer. No significant differences in the frequencies of total Tregs were detected in the spleens and lymph nodes in each of the treatment groups (Fig. 4B). In the blood, compared to non-transgenic mice, untreated APP/PS1 mice showed reduced, but not significant, total Treg frequencies. Notably, APP/PS1 mice treated with polyclonal Tregs showed significantly increased frequencies of total Tregs compared to untreated APP/PS1 mice. However, APP/PS1 mice treated with TCR_Aβ_- or EV-Tregs showed no significant differences in total Treg frequencies. Interestingly, total Tregs in the brain showed a significant increase in APP/PS1 mice treated with TCR_Aβ_-Tregs as compared to all treatment groups. Moreover, mice treated with control EV-Tregs showed significant reduction of total Tregs compared to all groups. Taken together, treatment with TCR_Aβ_-, polyclonal-, or EV-Tregs showed little or no effect on the distribution of total Tregs in the spleen, lymph node, and blood suggesting most Tregs remain predominantly in the systemic circulation, whereas treatment with TCR_Aβ_-Tregs owing to their Aβ reactivity, easily infiltrate the brain as it is a major site of pathological amyloid deposition and neuroinflammation.

To further confirm the brain-targeting efficacy of engineered Tregs, we adoptively transferred ^18^F-FDG radiolabeled wild-type polyclonal Tregs or TCR_Aβ_-Tregs to APP/PS1 mice and evaluated their biodistribution by PET imaging at 0.5, 2, 4, and 6 h after transfer. Interestingly, compared to wild-type Tregs, TCR_Aβ_-Tregs yielded significantly higher signal in the brain 0.5-h post-transfer (Fig. 4C and D). Increased TCR_Aβ_-Treg infiltration into the brain was sustained for up to 4–6 h. Together, this data highlights more efficient brain-targeting of TCR_Aβ_-Tregs compared to polyclonal Tregs.

### TCR_Aβ_-Tregs and systemic immune function

Tregs play a major role in maintaining immune tolerance and Treg dysfunction has been implicated in progression of AD pathology [29, 54, 55]. Studies have shown a protective role of ex vivo expanded Tregs in AD [28, 29, 56]. Herein, we evaluated changes in the suppressive function of peripheral Tregs after adoptive transfer of TCR_Aβ_-, polyclonal-, or EV-Tregs to APP/PS1 recipients. Compared to Tregs from untreated APP/PS1 mice, Tregs from APP/PS1 mice treated with TCR_Aβ_-Tregs or polyclonal Tregs showed elevated suppressive function with TCR_Aβ_-Tregs inducing the highest suppression (Fig. 4E). In contrast, Tregs from mice receiving EV-Tregs were functionally like untreated APP/PS1 mice.

Further, given the transient nature of TCR_Aβ_ expression by TCR_Aβ_-Tregs, we examined TCR_Aβ_ expression after adoptive transfer TCR_Aβ_-, polyclonal-, or EV-Tregs to APP/PS1 recipient animals. To perform these evaluations, splenocytes isolated at the time of sacrifice 2 weeks after the last transfer were stimulated with Aβ_42_ peptide in the presence of feeder cells (irradiated splenocytes 1:5 ratio) and low dose IL-2 (20U/mL) for a week. Post-stimulation, cells were stained with fluorescently labeled Aβ-tetramer (MHCII-IA^b^–KLVFFAEDVG-SNKGA) to evaluate the frequency of TCR_Aβ_-reactive CD4+ CD25+ Tregs. As expected, due to the presence of amyloid-β deposits in the AD mouse model, TCR_Aβ_ reactive Tregs were observed in untreated APP/PS1 mice at higher levels than found in non-transgenic mice. (Fig. 4F). However, only APP/PS1 mice treated with TCR_Aβ_-Tregs showed significant increase in the frequency of Aβ tetramer reactive Tregs (CD4+ CD25+) compared to untreated APP/PS1 mice or mice treated with polyclonal Tregs or EV-Tregs. Treatment of APP/PS1 mice with polyclonal Tregs or EV-Tregs induced no significant increases in frequencies of Aβ tetramer reactive Tregs. Additionally, there was no significant changes in Aβ tetramer reactive CD4+ CD25- cell populations in all treatments compared against APP/PS1 mice.

### TCR_Aβ_-Tregs reduce the amyloid burden

**Fig. 5:**
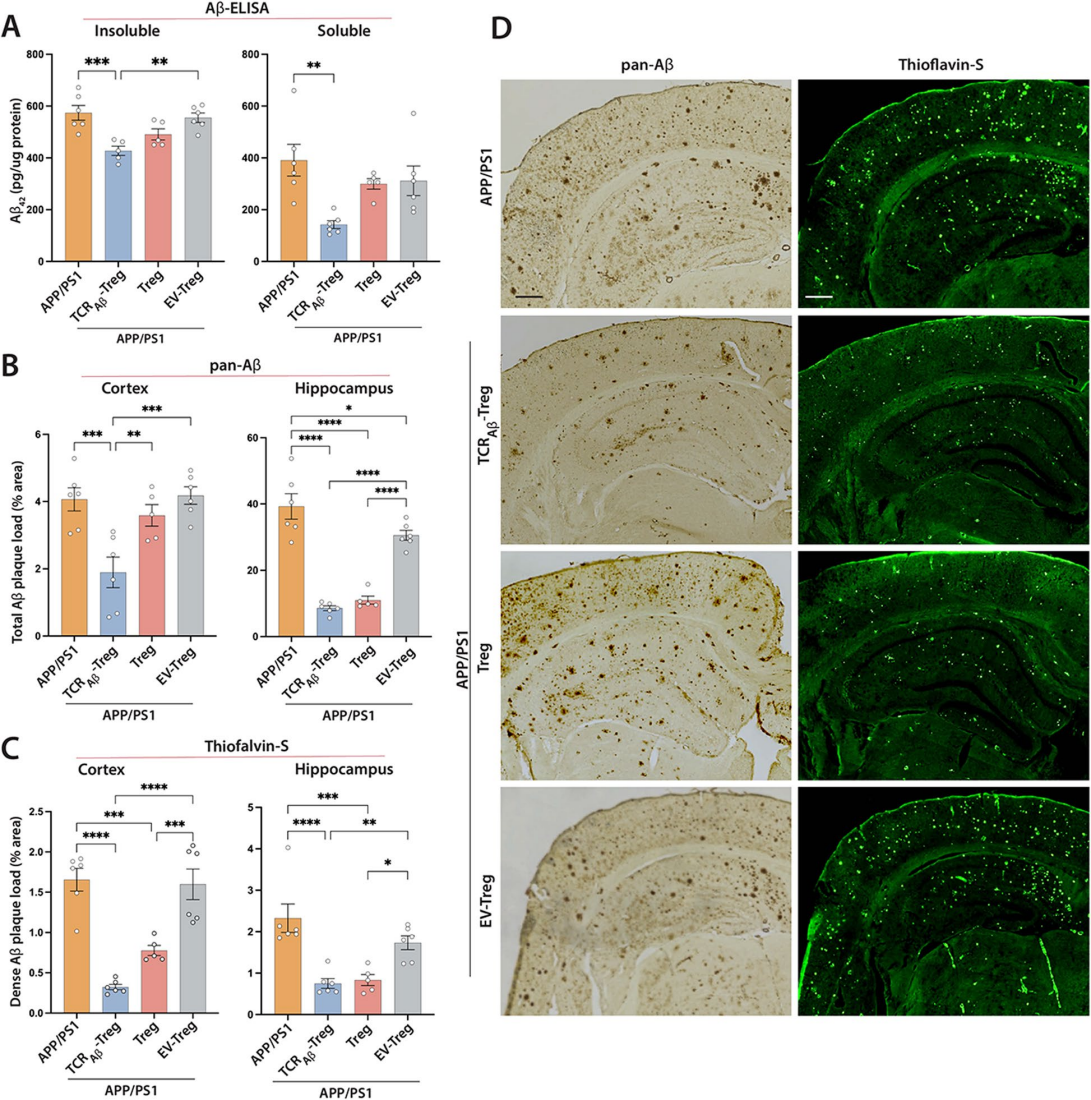
*Adoptive transfer of TCR*_*Aβ*_*-Tregs reduces amyloid load in APP/PS1 mice*. APP/PS1 mice were untreated or treated with TCR_Aβ_-Tregs, polyclonal Tregs (Treg) or EV-Tregs (TCR^−−^-Tregs electroporated with empty plasmid vector) by adoptive transfer and brain tissues acquired 3 weeks post adoptive transfer **A**. ELISA performed to quantify Aβ_1–42_ levels in the brain using Tris–HCl (soluble) and guanidine-HCL (insoluble) fractions of cortical tissue. **B**,** C**. ImmunohistoFchemistry (pan-Aβ) and immunofluorescence (Thioflavin-S) to determine the area occupied by insoluble Aβ plaques in cortical and hippocampal regions. **D** Representative images showing amyloid plaque (pan-Aβ) and Thioflavin-S staining in different brain regions. Percent area occupied was quantified using Cavalieri estimator probe. Scale bar = 100 µm. **A-C** Data presented as mean ± SEM for *n* = 5–6 mice/group. One-way ANOVA followed by Turkey’s post hoc test was used to determine significant differences between experimental groups. *p < 0.05, **p < 0.01, ***p < 0.001, ****p < *0.0001*

We next evaluated the effect of TCR_Aβ_-Tregs on the amyloid burden in cortex and hippocampus of AD mice. While APP/PS1 mice show significant amyloid deposits and loads, adoptive transfer of TCR_Aβ_-Tregs reduced both soluble and insoluble fragments of Aβ in the cortex, whereas polyclonal Treg treatment slightly, but insignificantly, reduced Aβ deposition (Fig. 5A). Treatment with EV-Tregs did not significantly affect amyloid load compared to untreated APP/PS1 mice. We then evaluated Treg-mediated effects on amyloid plaque deposition in the mice brain by immunohistochemistry. Treatment with TCR_Aβ_-Tregs reduced amyloid plaque in the cortex compared to untreated APP/PS1 mice and adoptive transfer of TCR_Aβ_-Tregs or polyclonal Tregs reduced total Aβ plaques in hippocampal tissues as determined by pan-Aβ staining (Fig. 5B and D). Treatment with EV-Tregs showed no significant effects in either cortex or hippocampus. We next determined the effects of Tregs on brain area occupied by dense amyloid plaques using Thioflavin-S immunohistochemistry. Adoptive transfer of either TCR_Aβ_-Tregs or polyclonal Tregs to APP/PS1 mice reduced dense amyloid plaque deposition in both the cortex and hippocampus compared with untreated APP/PS1 mice (Fig. 5C and D). Treatment with EV-Tregs did not show significant differences in cortical or hippocampal tissues compared to those tissues of APP/PS1 mice.

### TCR_A__β_-Tregs reduce reactive microglia

**Fig. 6:**
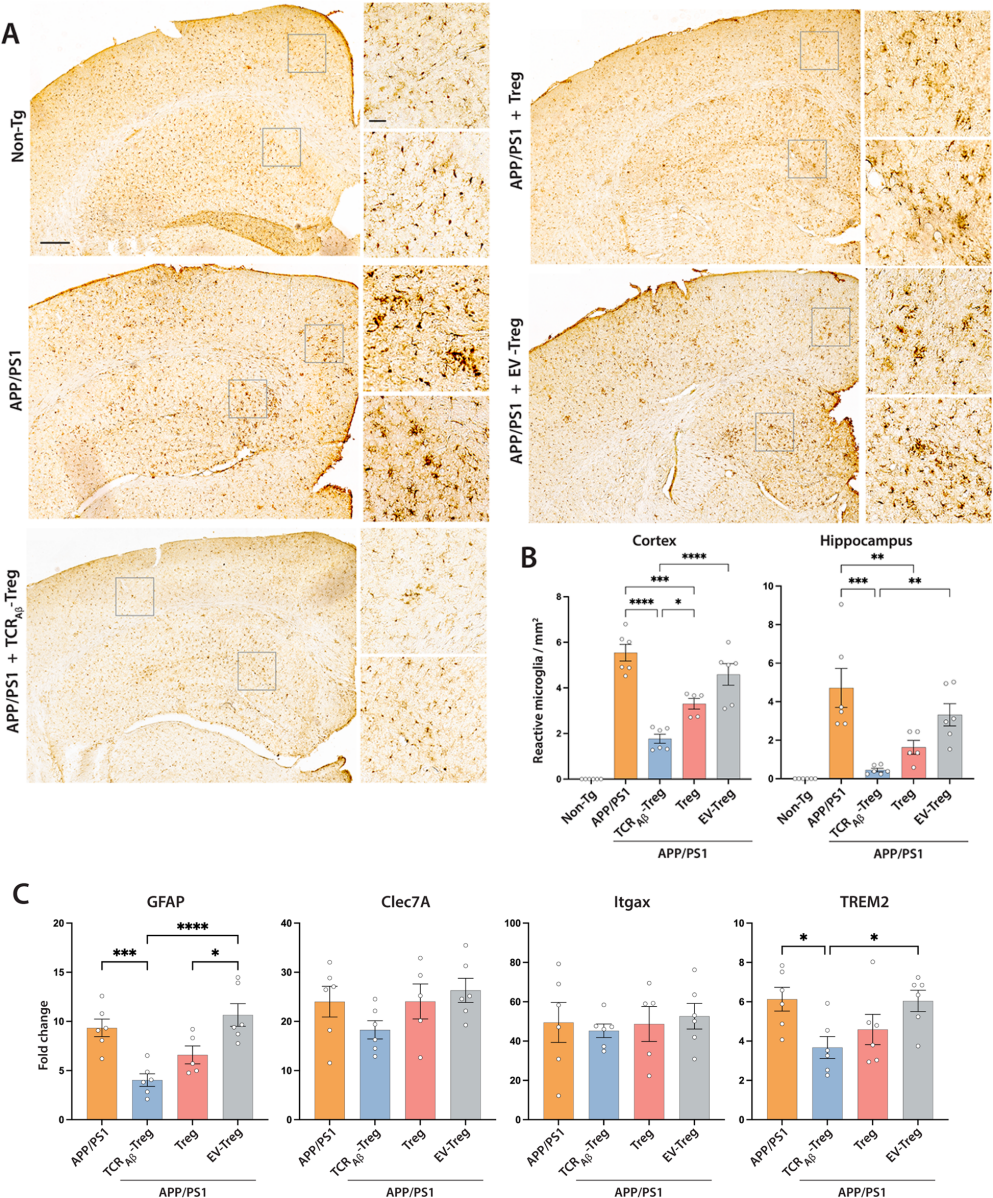
*Adoptive transfer of TCR*_*Aβ*_*-Tregs reduces reactive microglia in APP/PS1 mice.* **A** APP/PS1 mice were untreated or treated with TCR_Aβ_-Tregs, polyclonal Tregs (Treg) or EV-Tregs (TCR^−−^-Tregs electroporated with empty plasmid vector) by adoptive transfer and brain tissues acquired 3 weeks post-transfer. Untreated non-transgenic mice served as controls. Representative images showing Iba1 reactive cells in brain regions. Scale bar = 100 µm. Areas with most Iba1+ reactive microglia are highlighted by inserts for the cortex and hippocampus. Scale bar = 50 µm. **B** Number of Iba1+ reactive microglia were quantified from immunohistochemistry in cortex and hippocampus using Optical Fractionator probe of Stereo Investigator. Data presented as mean ± SEM for 5–6 mice per group. **C** Changes in the expression of the disease-associated genes for astrocytes (GFAP) and microglia (Clec7A, Itgax, and TREM2) in cortical tissue by qPCR. Obtained CT values were normalized against the RPLP0 gene and non-Tg mice was used as control. Data presented as mean ± SEM. One-way ANOVA followed by Turkey’s post hoc test was used to determine significant differences between experimental groups. *p < 0.05, **p < 0.01, ***p < 0.001, ****p < *0.0001*

Microglia activation is a common hallmark of neuroinflammation observed in AD patients and animal models [57]. To determine the effects of TCR_Aβ_-Tregs on reactive microglial responses, we counted Iba-1-reactive cells with amoeboid morphology in cortex and hippocampus after adoptive transfer of TCR_Aβ_-, polyclonal-, or EV-Tregs to APP/PS1 recipients. Immunohistochemistry visually showed a remarkable increase in the number Iba1 positive (Iba1+) amoeboid cells in cortical and hippocampal tissues of untreated APP/PS1 mice compared to non-transgenic mice suggesting increased microglia activation in AD mice (Fig. 6A). Compared with untreated AD mice, treatment with TCR_Aβ_-Tregs or polyclonal Tregs reduced numbers of Iba1 + reactive microglia in cortices and hippocampi of APP/PS1 AD mice with greater reductions in cortical tissues produced by TCR_Aβ_-Tregs (Fig. 6A and B). Treatment with EV-Tregs yielded no significant reductions of reactive microglia numbers in either the hippocampus or the cortex of APP/PS1 mice. To further characterize the microglial signature after TCR_Aβ_-Treg treatment, we evaluated the transcriptional changes in disease-associated microglia (DAM) markers such as Clec7A, Itgax and TREM2 [58]. In line with the reduced reactive microglial phenotype, we observed decreased TREM2 expression with both polyclonal Treg and TCR_Aβ_-Treg treatments (Fig. 6C). However, only TCR_Aβ_-Treg treatments reached significance compared against both untreated and EV-Treg treated APP/PS1 mice. Additionally, trends in Clec7A and Itgax expressions were recorded but without significant changes seen following TCR_Aβ_-Treg treatments (Fig. 6C). In addition to microglia, astrocytes have been implicated in promoting neuroinflammation in Alzheimer’s disease [59, 60]. Reactive astrocytes were assessed by evaluating GFAP expression. Both Treg and TCR_Aβ_-Treg treatments showed significantly reduced GFAP expression. However, only TCR_Aβ_-Treg treatments show the highest reductions compared to untreated and EV-Treg treated APP/PS1 mice (Fig. 6C).

## Discussion

We posit that Aβ-specific Tregs (TCR_Aβ_-Treg) can be a disease modifying therapy for AD. In this study, we compared the neuroprotective efficacy of TCR_Aβ_-Treg against polyclonal Treg as a treatment strategy in the APP/PS1 mouse model for AD. The Aβ-Treg cells were engineered using the TCR_Aβ_ identified from Aβ-reactive monoclonal Teff cells [41]. The antigen specificity was shown to enhance Treg-mediated neuroprotective responses. Comparisons between TCR_Aβ_-Tregs and polyclonal Tregs demonstrated improved neuroprotective responses which were defined by reduction of reactive microglia, diminished amyloid deposition, and improved memory formation. Notably, compared to the diffuse systemic distribution of polyclonal Tregs, TCR_Aβ_-Tregs specifically accumulated into the brain and elicit anti-inflammatory responses. The TCR_Aβ_-Tregs exerted anti-inflammatory activities by both bystander and antigen-mediated immunosuppressive activities.

These results fulfil an unmet need as recent FDA-approved antibody therapies for AD have met with mixed success [61]. This allows an increased interest in developing T-cell therapies for AD. Notably, prior studies have identified both CD4 + and CD8 + T cell subsets with either pro-inflammatory or regulatory activities which have yielded mixed disease outcomes for neurodegenerative disorders. While the CD4 + T cell subsets have well defined cell surface markers, transcription factors, and cytokines to categorize them into subtypes such as Th1, Th17, and Treg; the CD8+ T cell effects on disease remains poorly defined [62]. Prior reports demonstrated an AD immune signature of increased numbers of CD8+ T effector memory CD45RA+ (T_EMRA_) cells negatively associated with cognition and single-cell RNA sequencing showed their effect on TCR signaling [22, 62, 63]. In contrast works from our own laboratory have demonstrated a functional role for CD4+ Tregs in the control of neuroinflammation and affecting neuronal repair [37, 38, 40]. CD4+ CD25+ Foxp3+ Tregs have been shown to control Teff immune responses through restraint of T cell activation. This leads to the maintenance of brain tissue homeostasis and repair and shown to be effective in a broad range of neurodegenerative diseases that include amyotrophic lateral sclerosis, stroke, PD, and AD [28, 35–40]. Brain-resident CD69+ Tregs are present in the healthy brain with rapid expansion observed during neuroinflammatory processes serving to control astrogliosis by amphiregulin and shifting microglia into neuroprotective signatures through IL-10 [64, 65]. Tregs, on the other hand, have been shown to have a beneficial role in preclinical mouse models of AD. Transient depletion of Tregs accelerate cognitive decline, whereas amplification of Tregs including the use of low dose IL-2 improve cognitive outcomes in the same APP/PS1 mice used in this report [36]. Further studies have shown that induction of Tregs using therapeutic agents or adoptive transfer of polyclonal Tregs is neuroprotective outcomes against AD, PD [28, 36, 38, 39] and multiple sclerosis (MS) [66]. The safety and feasibility of a polyclonal Treg therapy have been established by numerous clinical trials in the field of autoimmune diseases and transplant rejection [67]. However, polyclonal Treg treatments rely on the bystander effect from Tregs homing into different tissues in an antigen-independent fashion resulting in global immune suppression [68]. Preclinical studies in autoimmune diseases indicate that antigen-specific Tregs could be more efficient by controlling pathological immune responses in a disease specific manner [69–73]. Antigen-specific Tregs migrate and accumulate at the site of cognate antigen expression where they exert both bystander and antigen-specific immune responses, and reduce complications associated with broad immunosuppression [69, 74]. This can be advantageous in AD where Treg access to diseased brain regions is required for neuroprotective and anti-inflammatory responses.

Identifying endogenous disease reactive Tregs or developing them though immunization is further complicated by low Treg precursor frequencies and lack of effective expansions. Recently, putative Aβ-reactive Tregs were generated by Aβ immunization of Treg depleted mice [31]. However, this approach however is limited by the fact that the Tregs generated are not monoclonal due to the low precursor frequencies of antigen reactive Treg post immunization [75]. Additionally, the lack of comparisons made against polyclonal Treg treatment in the study complicates the interpretation of the potential advantage of antigen specific Treg therapy. Current efforts at developing antigen-specific Tregs rely on transducing antigen-specific TCRs identified from Teff cells into Treg cells [76, 77]. Although, this approach was effectively used in manipulating human Treg cells, mouse Treg cells are extremely difficult to transduce [78]. Notably, our TCR_Aβ_ lentiviral constructs while successfully transducing CEMSS, 3T3 and human PBMC’s with the TCR_Aβ_, were less effective at transducing mouse Tregs. To overcome this limitation, we generated TCR_Aβ_-Tregs that transiently express an Aβ-specific TCR by electroporation of a plasmid encoding the TCR_Aβ_. The rationale behind this approach is that in a therapeutic setting, adoptively transferred Tregs need not persist indefinitely, but long enough to confer suppressive capacity to other immune cells located at the affected tissue via a phenomenon called ‘infectious tolerance’ [79, 80]. Therefore, Treg cells transiently expressing TCR_Aβ_ will generate the necessary proof-of-concept response for developing human TCR_Aβ_-Tregs for therapeutic evaluation.

In the current study, monoclonal TCR_Aβ_-Treg were generated by incorporating the Aβ-specific TCR identified from a highly reactive monoclonal Aβ-Teff cell previously developed in our lab [41]. Splenic Tregs were isolated from mice with homozygous MHC background (B6;129) and endogenous TCRs were eliminated using CRISPR-Cas9 technology to avoid nonspecific immune reactions. Electroporation of the plasmid encoding TCR_Aβ_ generated antigen-specific Tregs that transiently expressed the Aβ-specific TCR. Aβ-specificity of the engineered TCR_Aβ_-Tregs was demonstrated by recognition by an MHC-Aβ-peptide tetramer. TCR_Aβ_-Tregs showed significantly higher immune suppression owing to the transfer of highly reactive TCR_Aβ_. However, Tregs can show dominant bystander effect, where they suppress Teffs in an antigen non-specific manner [81]. Aβ-specific immunosuppressive function of the engineered TCR_Aβ_-Tregs was shown when MHC-Aβ-tetramer was used as the Treg stimulant. Further evaluation of the cytokine profile shows that compared to polyclonal Tregs, TCR_Aβ_-Tregs expressed decreased proinflammatory cytokines such as IFN-γ [28] and increased granulocyte–macrophage colony-stimulating factor (GM-CSF). This serves to support their abilities to elicit Treg differentiation from naïve T cells by tolerogenic dendritic cells which serve to polarize T cells toward Th2 phenotypes [82]. Additionally, increased production of IL-4 supports TCR_Aβ_-Treg immunosuppressive functions of IFN-γ secreting CD4+ T cells [83] while CCL2 and CCL5 are potential chemokines in Treg recruitment in vivo [84]. Our data suggests that incorporating the TCR identified by disease-reactive Teffs is a viable strategy for engineering antigen-specific Tregs that are immunosuppressive in an antigen driven manner.

In a healthy brain, infiltration of peripheral lymphocytes is well-controlled [85]. However, with AD progression, the Aβ lymphatic drainage is compromised and leads to increased infiltration of peripheral immune cells that exacerbate AD pathology [85]. The central hypothesis behind developing antigen-specific Treg therapies for AD is to target Aβ-specific cells to amyloid-rich brain sites. In the current study, we show that in APP/PS1 mice, adoptively transferred TCR_Aβ_-Tregs significantly infiltrate the brain, while transferred polyclonal Tregs predominantly remain in the peripheral circulation. ^18^F-FDG radiolabeled cell tracking highlights the brain targeting efficacy of TCR_Aβ_-Treg in an antigen-dependent manner. Although, TCR_Aβ_-Tregs targeted the brain in APP/PS1 mice, they were able to significantly increase systemic Treg function owing to the high affinity TCR_Aβ_ incorporated into the cells. Notably, even though the engineered TCR_Aβ_-Tregs only transiently expressed the Aβ-specific TCR, stimulation of Tregs with human-Aβ peptide showed higher MHC-Aβ-tetramer reactive Tregs in mice treated with TCR_Aβ_-Tregs. These data suggested disease-specific priming of naïve Tregs. Cognitive function evaluated by ^18^F-FDG PET is considered an imaging biomarker for AD [86–88]. Decreased ^18^F-FDG uptake represents a reduction in neuronal energy demand mainly arising from synaptic loss caused by amyloid pathology in patients. Adoptive transfer of TCR_Aβ_-Tregs also increased brain glucose uptake compared to treatment with polyclonal Tregs. Notably, increased brain glucose uptake after TCR_Aβ_-Treg treatment correlated with improved memory outcomes in both RAWM and Y maze behavioral tests. Taken together, these results show that the TCR_Aβ_-Tregs, even though transiently expressing the TCR_Aβ_, were able to target the brain and improve brain function and memory outcomes.

Additionally, microglia serve a key role in processing and presenting self-antigens, including Aβ, to maintain immune tolerance [28, 89]. Non-activated microglia exhibit ramified morphology and can clear Aβ deposits through phagocytosis [45]. However, with disease progression microglia become more activated and acquire amoeboid morphology with compromised phagocytic capabilities and elevated neurotoxicity [35, 45]. Studies have shown that cerebral Tregs restrain microglial inflammatory responses, and treatment with ex vivo expanded polyclonal Tregs was shown to suppress microglial inflammation [30, 31, 90]. Our results show that TCR_Aβ_-Tregs are more effective than polyclonal Tregs at reducing reactive microglia both in the cortex and hippocampus. The brain targeting capacity of TCR_Aβ_-Tregs and subsequent increase in percentage of brain Tregs resulted in greater reduction in inflammatory phenotypes of microglia compared to polyclonal Treg treatment. In addition to transforming microglia to a non-reactive phenotype, our results show that TCR_Aβ_-Tregs are more effective at reducing amyloid deposition in both hippocampal and cortical regions of the brain as demonstrated by reduced soluble and insoluble Aβ_1-42_ load determined by ELISA and Aβ plaques quantitative IHC. Notably, compared to polyclonal Tregs, TCR_Aβ_-Tregs were more effective at reducing dense, pathological Thioflavin-S positive Aβ deposits. Overall, our results show that the brain-targeting efficacy and disease-specific immunosuppressive function of TCR_Aβ_-Tregs lead to improved reduction of neuroinflammatory reactive microglia which further enhance clearance of amyloid plaque.

In summary, the current findings demonstrate proof-of-concept preclinical data in which disease-specific TCR_Aβ_-Tregs are more effective at reducing amyloid pathology and improving cognitive outcomes than polyclonal Tregs. The transient expression of TCR_Aβ_ by engineered TCR_Aβ_-Tregs is a noted limitation in the study. However, the strong neuroprotective results shown in APP/PS1 mice despite the transient expression of the TCR_Aβ_ underlies the significant therapeutic potential achievable with ‘long-lived’ TCR_Aβ_-Tregs. Notably, the results confirm a clinically translatable strategy to develop stably expressing human TCR_Aβ_-Tregs. A second limitation is the absence of significant differences between TCR_Aβ_-Tregs and Treg treatments on the hippocampal amyloid burden while significant reductions were recorded in the cortex. We posit that hippocampal quantitation is limited by the amounts of tissue regions available for testing. Taken together, the data support the hypothesis that TCRs engineered as chimeric antigen receptors (CARs) will enable further development of Treg therapies for AD.

## Conclusion

This is a proof-of-concept study that confirms the feasibility of Aβ-specific Treg AD therapy. Treatment with TCR_Aβ_-Tregs in a relevant animal model enabled reductions in reactive microglia, amyloid load, and cognitive decline. Importantly, TCR_Aβ_-Tregs were demonstrated to target amyloid-rich regions in an AD-diseased brain while demonstrating antigen-specific immunosuppression. We conclude that Aβ-specific Tregs can be a translatable therapeutic approach for AD and perhaps other neurodegenerative diseases.

### Supplementary Information

**Additional file 1.**

## Data Availability

All data needed to evaluate the conclusions in the paper are included in the paper. The Aβ-TCR sequence used to engineer the Treg cells in the study is not publicly available due to the patent currently under review: Cell therapy for Alzheimer’s disease” (EFS ID: 42,475,225, Application number: 63175747, Docket number: 21084P).

## Abbreviations

AD: Alzheimer’s disease
PD: Parkinson’s disease
MS: Multiple Sclerosis
Aβ: Amyloid beta
TCR: T cell receptor
TCR^−−^-Treg: Endogenous TCR knocked out of Treg
TCR_Aβ_-Treg: Aβ antigen specific Treg
EV-Treg: TCR^−−^-Treg electroporated with empty plasmid vector
Treg: T regulatory cell
Teff: T effector cell
Tresp: T responder cell
Iba-1: Ionized calcium binding adaptor molecule 1
^18^F-FDG-PET: ^18^F-2-fluoro-deoxy- D-glucose positron emission tomography
