# Supplementary material for: Amyloid-β specific regulatory T cells attenuate Alzheimer’s disease pathobiology in APP/PS1 mice
Source: Mol Neurodegener. 2023 Dec 18;18:97. doi: 10.1186/s13024-023-00692-7 (PMC10729469; doi:10.1186/s13024-023-00692-7)
Supplement: Supplementary file 1 — Additional file 1. [file 13024_2023_692_MOESM1_ESM.docx]

*Supplementary Materials*

**Amyloid-β Specific Regulatory T Cells Attenuate Alzheimer’s Disease Pathobiology in APP/PS1 M­­ice**

## Pravin Yeapuri^1*^, Jatin Machhi^1*^, Yaman Lu^1^, Mai Mohamed Abdelmoaty^1^, Rana Kadry^1^, Milankumar Patel^1^, Shaurav Bhattarai^1^, Eugene Lu^1^, Krista L Namminga^1^, Katherine E. Olson^1^, Emma G. Foster^1^, R. Lee Mosley^1^, Howard E. Gendelman^1, #^

*^1^Department of Pharmacology and Experimental Neuroscience, College of Medicine, University of Nebraska Medical Center, NE 68198, USA*

*Contributed equally

^#^**Corresponding Author:** Howard E. Gendelman, Department of Pharmacology and Experimental Neuroscience, University of Nebraska Medical Center, Omaha, NE 6898-5880; phone 402-559-8920; fax 402-559-3744; email [hegendel@unmc.edu](mailto:hegendel@unmc.edu)

**Data Availability Statement**

All data generated, written up, or analyzed from this study are either included in this published article or its supplementary information files.


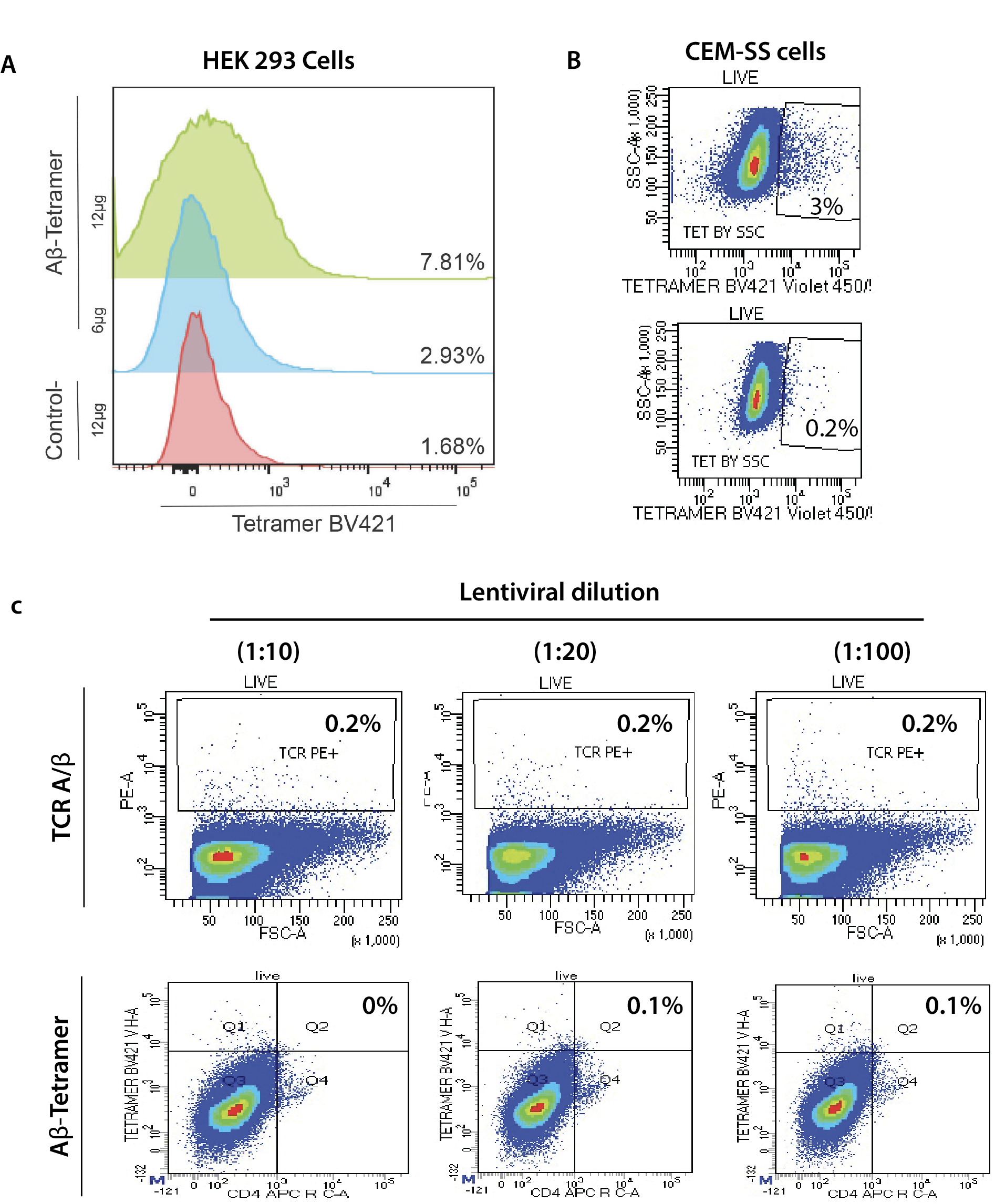


**Additional file 1: Figure S1.** ***Aβ-TCR Lentiviral transduction of HEK-293, CEM-SS T cells and TCR^--^-Treg***. **A**. Flow cytometry gating confirming the MHC-Aβ-Tetramer staining of HEK-293 cells transduced with TCR_Aβ_ lentiviral constructs. **B**. Flow cytometry gating confirming the MHC-Aβ-Tetramer staining of CEM-SS cells transduced with TCR_Aβ_ lentiviral constructs. **C**. Flowcytometry gating showing the inability of TCR_Aβ_ lentiviral constructs (10^9^ transduction units/mL) incubated at 1:10, 1:20 or 1:100 dilution using TransDux Max to transduce TCR^--^-Treg (Treg cells knocked out of endogenous TRC using CRISPR-Cas9 technology).


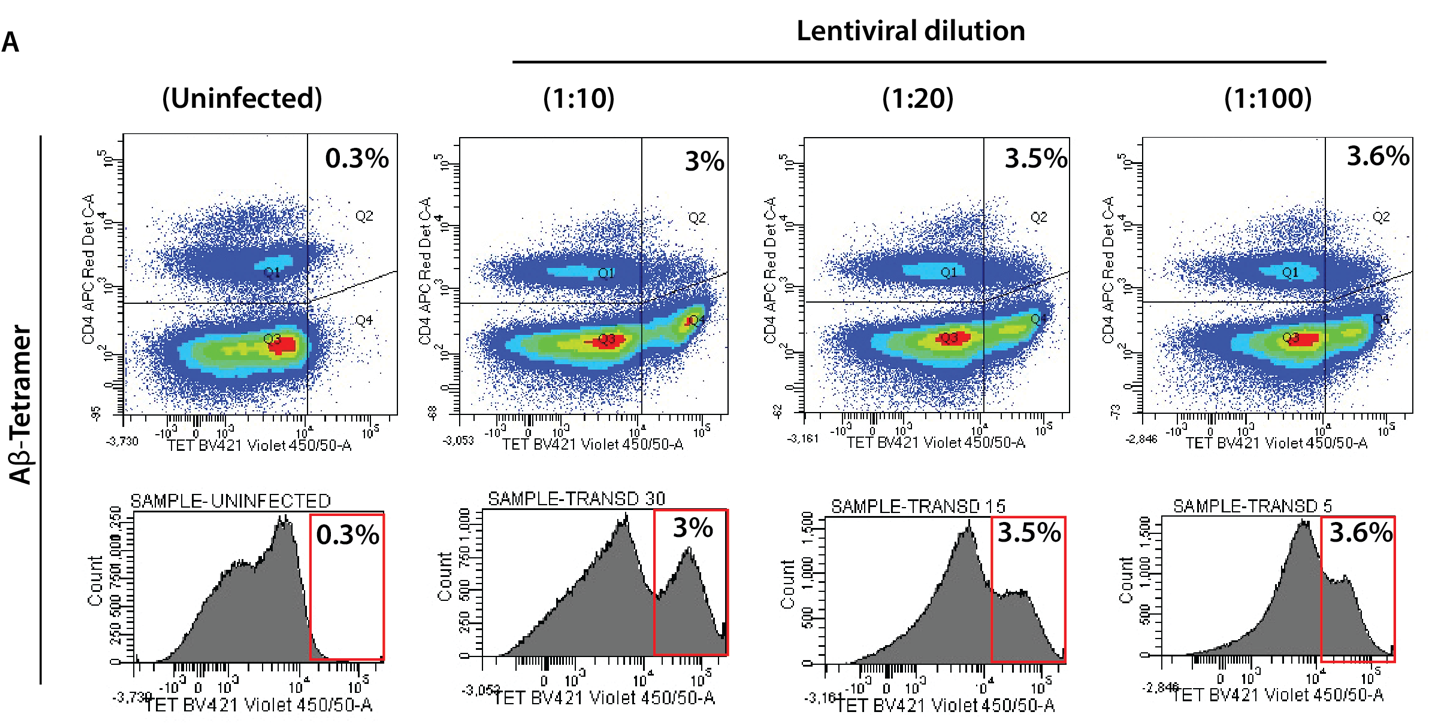


**Additional file 1: Figure S2*. Aβ-TCR Lentiviral transduction of Human PBMCs.* A**. Flowcytometry gating showing the TCR_Aβ_ lentiviral transduction (stock concentration=10^9^ transduction units/mL) of human PBMCs incubated at 1:10, 1:20 or 1:100 dilution using TransDux Max. Successful transduction of TCR_Aβ_ was confirmed by staining with MHC-Aβ-Tetramer compared to non-transduced cells.


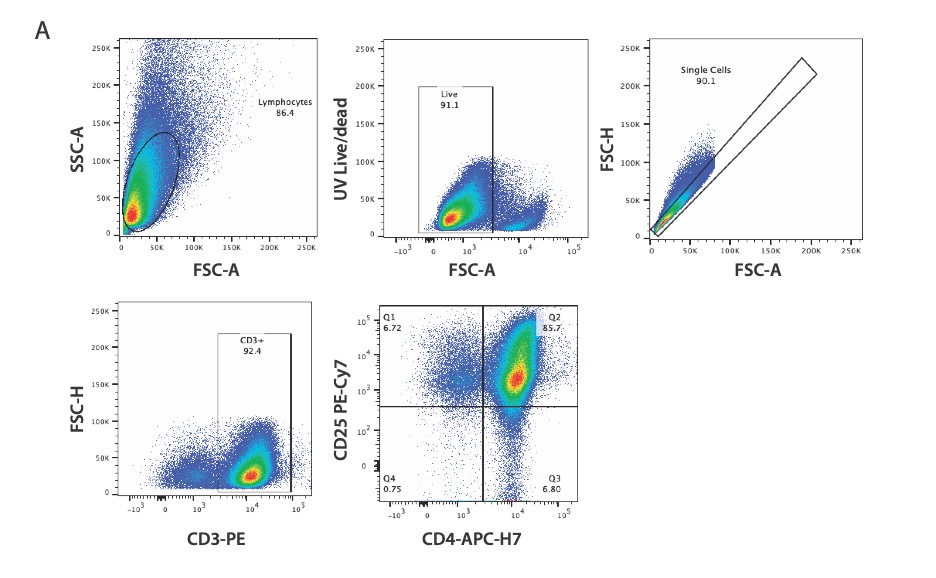


**Additional file 1: Figure S3*. Phenotype characterization of TCR_Aβ_-Tregs:* A**. Flowcytometry gating showing CD4+CD25+ Treg phenotype of TCR_Aβ_-Tregs on day 2 post electroporation of TCR^--^-Tregs (TCR knockout Tregs) with 0.5 μL of TCR_Aβ_ plasmid (plasmid conc.=1.5 μg/μL).

******

**Additional file 1: Supplementary Table 1*. Statistical analysis of cytokine profile.*** Polyclonal Tregs (Treg), TCR^--^-Tregs (TCR knockout Tregs), and TCR_Aβ_-Tregs (TCR^--^-Tregs + 0.5 μL TCR_Aβ_ plasmid) were stimulated with PMA and ionomycin and culture supernatants assessed for 40 cytokine concentrations by mouse cytokine array. Data presented as mean intensities ± SEM. Statistical significance of cytokine expression between experimental groups determined by two- way ANOVA followed by Turkey’s post hoc test was used to determine significant differences between experimental groups*. *p<0.05, **p<0.01, ***p<0.001, ****p<0.0001*.

| **Gene** | **Primer sequence (5’-3’)** | **Reference sequence** |
| --- | --- | --- |
| RPLP0 | F: TTATAACCCTGAAGTGCTCGAC  R: CGCTTGTACCCATTGATGATG  Probe: 56-FAM/AGGCCCTGC/ZEN/ACTCTCGCTT/3IABkFQ/ | NM_007475 |
| Clec7A | F:TTCAGCACTCAAGACATCCAT  R:CCACTACTACCACAAAGCACA  Probe: 56-FAM/TCTTCACCT /ZEN/TGGAGGCCCATTGC/3IABkFQ/ | NM_020008 |
| Itgax | F: GCTCTGCTTTCTACTGAGTTCA  R:CTACCCGAGCCATCAATCAG  Probe: 56-FAM/AGCCAGAAC /ZEN/TTCCCAACTGCACA/3IABkFQ/ | NM_021334 |
| GFAP | F: TGCAGGAGTACCAGGATCTAC  R:GATCTGGAGGTTGGAGAAAGTC  Probe: 56-FAM/ CAGGAATGG/ZEN/TGATGCGGTTTTCTTCG /3IABkFQ/ | NM_010277 |
| Trem2 | F: GACCTCTCCACCAGTTTCTC  R: GCTTCAAGGCGTCATAAGTACA  Probe: 56-FAM/TCCCAAGCC/ZEN/CTCAACACCACG/3IABkFQ/ | NM_031254 |

**Additional file 1: Supplementary Table 2*. Details of primers.***
